# Host Defense Peptides for Intracellular Bacterial Infections: Overcoming Translational Barriers Through Peptide Engineering and Nanotechnology-Based Drug Delivery Systems

**DOI:** 10.3390/pharmaceutics18101326

**Published:** 2026-10-20

**Authors:** Aswathy R. Devan, Pradeesh Babu, Geetha B. Kumar, Bipin G. Nair, Agiesh Kumar Balakrishna Pillai, Aravind Madhavan

**Affiliations:** 1 School of Biotechnology, Amrita Vishwa Vidyapeetham, Amritapuri, Kollam 690525, Kerala, India; 2 Department of Clinical Virology, Institute of Advanced Virology, Thiruvananthapuram 695317, Kerala, India

**Keywords:** intracellular bacteria, host immunity, host defense peptides, peptide engineering, targeted peptide delivery

## Abstract

Intracellular bacterial infections represent a major therapeutic challenge due to the ability of pathogens to persist within host cells, evade host immune surveillance and exhibit reduced susceptibility to conventional antibiotics. Consequently, these infections are often associated with severe clinical manifestations, antibiotic resistance, treatment failure, and increased mortality. Host-directed therapies have emerged as a promising approach by strengthening host defense mechanisms through the modulation of innate and adaptive immune responses, thereby enhancing the clearance of intracellular pathogens. Among these strategies, host defense peptides (HDPs) have gained considerable attention as they combine broad-spectrum antimicrobial activity with the ability to modulate host immune responses. HDPs also regulate the hyperinflammatory phase of infection, thereby limiting excessive tissue damage and infection-associated complications. Despite the promising preclinical efficacy against bacterial infections, the clinical translation of peptide-based therapeutics is hindered mainly by its inherent pharmacokinetic limitations, including poor stability, rapid proteolytic degradation, and limited bioavailability. However, recent advances in rational peptide engineering and nanotechnology-based delivery systems have shown considerable promise in overcoming these limitations. This review discusses the mechanisms employed by intracellular bacterial pathogens to colonize and survive within host cells. We highlight the immunomodulatory role of HDPs in controlling intracellular bacterial infections and critically examine the major barriers limiting their clinical translation. Finally, we summarize recent advances in peptide engineering and targeted delivery strategies that facilitate the development of HDP-based therapeutics for the effective treatment of intracellular bacterial infections.

## 1. Introduction

Infectious diseases continue to pose a major global health burden, with the world currently witnessing an era marked by recurrent major outbreaks and the emergence of new infectious threats. A study of global disease burden reported that, in 2019, approximately 704 million disability-adjusted life years (DALYs) were associated with 85 pathogens, of which nearly 59% were attributed to bacterial infections [1,2]. Notably, five major bacterial pathogens other than tuberculosis—*Staphylococcus aureus*, *Escherichia coli*, *Streptococcus pneumoniae*, *Klebsiella pneumoniae*, and *Pseudomonas aeruginosa*—accounted for more than 50% of infection-related deaths worldwide [3]. Among these, intracellular bacterial pathogens represent a particularly significant clinical challenge, contributing to severe conditions such as pneumonia, sepsis, and chronic and recurrent infections, ultimately leading to increased mortality. These pathogens possess the ability to evade host immune defenses and exhibit reduced susceptibility to conventional antimicrobial therapies through their capacity to survive and persist within host cells, particularly macrophages [4]. In this context, host cells act as “Trojan horses” for bacterial pathogens, providing a protective niche that facilitates their survival, colonization, and dissemination to non-infected tissues through multiple adaptive strategies [5].

Conventional antibiotics often exhibit limited efficacy against intracellular bacterial pathogens due to inadequate intracellular penetration, antibiotic inactivation within the host cellular environment, and reduced susceptibility of dormant intracellular bacterial populations. Furthermore, this inappropriate and prolonged use of antibiotics accelerates the emergence and spread of antimicrobial resistance [6,7]. In addition, intracellular pathogens can evade innate immune defense by impairing the microbicidal functions of macrophages and subsequently suppressing the activation of adaptive immune responses through diverse immune evasion mechanisms. Consequently, these pathogens establish a favorable immunosuppressed intracellular microenvironment that promotes their survival, facilitates the acquisition of essential nutrients from the host, and supports their replication and persistence within infected tissues [8,9]. Therefore, implementation of effective strategies to target intracellular bacterial infections is essential.

In recent years, host-directed therapies (HDTs) have been extensively investigated as promising approaches for the treatment of intracellular bacterial infections, either alone or in combination with conventional antibiotics. These therapies enhance host defense mechanisms by modulating innate and adaptive immune responses, thereby facilitating the effective clearance of intracellular pathogens. Diverse therapeutic agents, including immunomodulators, repurposed drugs, vitamins, biologics, and phytochemicals, are currently being evaluated as HDTs in both preclinical and clinical studies [10]. Among these, antimicrobial peptides (AMPs), also known as host defense peptides (HDPs), have gained considerable attention because of their broad-spectrum antimicrobial and immunomodulatory activities [11,12]. HDPs are generally short peptides composed of approximately 10–50 amino acids and are widely distributed in microorganisms, plants, and animals. These peptides are produced either through ribosomal synthesis as part of the innate immune response or via non-ribosomal peptide synthesis pathways. In addition to their direct antimicrobial activity against a wide range of pathogens, HDPs can modulate host immune responses by enhancing phagocytosis, regulating autophagy, modulating inflammatory signaling, and promoting adaptive immune activation. These combined antimicrobial and immunomodulatory properties make HDPs promising therapeutic candidates for the management of intracellular bacterial infections [13].

Despite their promising therapeutic potential, the clinical application of HDPs remains limited due to poor pharmacokinetic properties, including instability, susceptibility to enzymatic degradation, and limited bioavailability. Recent advances in peptide engineering and nanotechnology have provided effective strategies to overcome these limitations by improving peptide stability, optimizing pharmacokinetic properties, and enabling site-specific delivery. Furthermore, the development of engineered or synthetic HDPs, together with nanocarrier-based delivery systems, significantly improves the translational potential of HDP-based therapies for intracellular bacterial infections [14,15].

In this review, we discuss the therapeutic potential of host defense peptides against intracellular bacterial infections, with particular emphasis on peptide engineering and nanotechnology-based approaches that may facilitate their clinical translation.

## 2. Intracellular Bacteria: Colonization and Survival Mechanisms Inside the Macrophages

### 2.1. Intracellular Bacterial Pathogens

Intracellular bacterial pathogens are broadly classified into obligate and facultative intracellular bacteria based on their dependence on the host cellular environment for survival and replication. Obligate intracellular bacteria function as cellular parasites, relying extensively on host metabolic machinery and nutrients to sustain their growth and propagation; thus, they are unable to replicate outside host cells. In contrast, facultative intracellular bacteria can survive in extracellular environments but also possess the ability to invade and persist within host cells, particularly macrophages. Intracellular localization enables these pathogens to evade innate immune defense, limit exposure to antimicrobial agents, and thereby promote bacterial survival and persistence within the host [16,17].

Most pathogenic obligate intracellular bacteria belong to the genera *Anaplasma*, *Coxiella*, *Chlamydia*, *Ehrlichia*, *Orientia*, *Rickettsia*, and *Simkania*. These pathogens exhibit specific cellular tropism and preferentially infect distinct host cell types, including epithelial cells (*Chlamydia*), endothelial cells (*Rickettsia*), neutrophils (*Anaplasma*), and monocytes or macrophages (*Ehrlichia*) [16]. In contrast, facultative intracellular pathogens such as *Mycobacterium tuberculosis*, *Salmonella enterica*, *Listeria monocytogenes*, *Brucella* spp., *Legionella pneumophila*, *Staphylococcus aureus*, and many others are frequently internalized by macrophages as part of the normal host defense process. However, instead of being eliminated, these pathogens adapt to the intracellular environment and employ diverse mechanisms to survive and persist within host cells [18,19].

Treatment of obligate intracellular bacterial infections primarily relies on pathogen-specific antibiotics capable of efficiently penetrating host cells and retaining activity within intracellular compartments. However, management of infections caused by facultative intracellular bacteria remains more challenging, as these pathogens can exist in both extracellular and intracellular forms, develop dormant populations, evade host immune responses, and exhibit multidrug resistance. Consequently, conventional antibiotic therapy often results in suboptimal clinical outcomes and incomplete bacterial clearance [18,19,20] (Table 1).

### 2.2. Innate Immune Functions of Macrophages in Bacterial Infection

Most intracellular bacterial pathogens preferentially target myeloid cells, particularly macrophages, which serve as professional phagocytes and important sentinels of innate immunity. Macrophages are widely distributed across various tissues, including Kupffer cells in the liver, alveolar macrophages in the lungs, microglia in the central nervous system, osteoclasts in bone, histiocytes in connective tissue, and Hofbauer cells in the placenta. Through continuous surveillance of their local microenvironment, macrophages recognize invading pathogens and initiate antimicrobial and inflammatory responses [9,36].

Macrophages express a broad repertoire of pattern recognition receptors (PRRs), including Toll-like receptors (TLRs), NOD-like receptors (NLRs), mannose receptors, C-type lectin receptors such as dectin-1, and scavenger receptors. These receptors recognize pathogen-associated molecular patterns (PAMPs) expressed by diverse microorganisms. Among these, dectin-1 and the mannose receptor (CD206) are important phagocytic receptors that recognize microbial surface components present on bacteria, fungi, and viruses. The interaction between PAMPs and PRRs triggers rearrangement of the phagocyte plasma membrane, leading to the formation of a phagocytic cup that engulfs the pathogen to form a phagosome. The phagosome subsequently matures and fuses with lysosomes to generate a phagolysosome. This phagolysosomal compartment creates an acidic microenvironment that activates degradative enzymes, antimicrobial peptides, and reactive oxygen species (ROS), which collectively mediate the killing and degradation of engulfed microorganisms [9,37].

Beyond direct microbial killing, macrophages coordinate local and systemic immune responses through the production of cytokines and chemokines. They also function as antigen-presenting cells by processing microbial antigens and presenting them through major histocompatibility complex (MHC) class II molecules to CD4+ T cells. Furthermore, IL-12 produced by activated macrophages promotes the differentiation of naïve T cells into Th1 cells. Thus, macrophages provide an important link between innate and adaptive immunity through pathogen recognition, phagocytosis, cytokine production, antigen presentation, and T-cell activation (Figure 1) [38,39].

Importantly, phenotypic plasticity of macrophages is a crucial factor regulating the immune response to bacterial infection. Macrophages recruited to infected tissue react to various stimuli and acquire either a classical or alternative phenotype. Classically activated or M1-like macrophages, induced by stimuli such as IFN-γ, LPS, and TNF, exhibit enhanced antimicrobial activity and produce ROS, nitric oxide (NO), and pro-inflammatory cytokines, including TNF-α, IL-1β, IL-6, and IL-12. In contrast, alternatively activated or M2-like macrophages can be induced by signals including IL-4, IL-10, and TGF-β and exert anti-inflammatory responses, tissue repair, and resolution of inflammation. This functional plasticity is particularly relevant to intracellular bacterial infections, as pathogens can manipulate macrophage activation and intracellular trafficking to establish a permissive niche for persistence and replication [40,41,42].

### 2.3. Subversion of Macrophage Defense by Intracellular Bacteria

Despite the potent microbicidal activity of macrophages, several bacterial pathogens, particularly intracellular bacteria, have evolved sophisticated survival strategies to evade macrophage-mediated killing and establish a protected intracellular niche that supports their replication and persistence.

#### 2.3.1. Surface Modifications to Evade Macrophage Recognition

Intracellular bacteria can modify or mask their surface structures, thereby reducing recognition by pattern recognition receptors (PRRs) on macrophages [43]. Lipid A, the hydrophobic component of lipopolysaccharide (LPS), is a key PAMP recognized by the TLR4/MD-2 receptor complex on immune cells. Binding of lipid A to TLR4/MD-2 activates downstream innate immune signaling, resulting in the production of pro-inflammatory mediators and the upregulation of co-stimulatory molecules that contribute to the initiation of adaptive immune responses. To evade this process, intracellular pathogens, including *Salmonella*, *Escherichia coli*, and *Yersinia* spp., modify the structure of lipid A, thereby altering TLR4 activation and attenuating inflammatory cytokine production [44,45]. Similarly, modification of peptidoglycan through acetylation or deacetylation can reduce the release of immunostimulatory peptidoglycan fragments, thereby limiting their recognition by NOD-like receptors [46]. Modification of the O-antigen represents another strategy by which bacteria alter the accessibility and antigenic properties of surface LPS, potentially reducing recognition by immune cells. This mechanism is particularly relevant to hypervirulent *Klebsiella pneumoniae*, in which capsule-mediated masking of the O-antigen has been reported to contribute to evasion of macrophage recognition and killing [47].

#### 2.3.2. Secretion of Virulence Factors to Promote Intracellular Survival

Bacterial protein secretion systems deliver effector proteins and other macromolecules directly into host cells, thereby modulating critical host cell processes such as vesicular trafficking, phagosome maturation, cytoskeletal dynamics, and immune signaling pathways [48]. Among the Type I-X1 bacterial secretion systems, Type III (T3SS), Type IV (T4SS), Type VI (T6SS), and Type VII (T7SS) secretion systems are mostly implicated in intracellular survival. The Type III secretion system is commonly utilized by pathogens such *as Salmonella enterica* [49], *Shigella flexneri*, and *Chlamydia* spp. [50], and *Yersinia* spp. [51]. This system facilitates “trigger”-mediated bacterial internalization, intracellular vacuolar persistence, cytosolic escape, and intercellular dissemination through the injection of virulence effector proteins into host cells [52]. The Type IV secretion system is employed by several clinically important intracellular pathogens, including *Brucella suis* (VirB system) [53], *Coxiella burnetii* [54], *Anaplasma phagocytophilum* [55], and *Ehrlichia chaffeensis* [56]. A distinctive feature of the T4SS is its ability to transport both proteins and nucleic acids into eukaryotic host cells, thereby enabling extensive modulation of host cellular functions and intracellular trafficking pathways [57]. The Type VI secretion system functions through a bacteriophage-like injection mechanism that delivers effector proteins into eukaryotic host cells as well as competing bacterial cells [58]. In *Salmonella* spp., the T6SS contributes significantly to intracellular survival, virulence, and bacterial competition within the host environment [59]. In contrast, *Mycobacterium* spp. predominantly utilizes the Type VII secretion system (T7SS), particularly the ESX secretion machinery, to mediate intracellular survival and pathogenesis. Through this system, effector proteins such as ESAT-6 and CFP-10 are secreted across the mycobacterial cell envelope, promoting phagosomal membrane disruption, cytosolic access, immune modulation, and persistence within macrophages [60,61].

#### 2.3.3. Manipulation of Host Signaling and Macrophage Activation

Macrophage activation, polarization, and functions are regulated by interconnected pattern recognition receptors (PRRs) and cytokine-mediated signaling pathways. TLRs, NLRs, and the cGAS–STING pathway facilitate pathogen recognition and initiate innate immune responses, while cytokines such as TNF-α, IFN-γ, IL-6, and IL-10 regulate macrophage activity through NF-κB, MAPK, and JAK–STAT pathways. In addition, CSF-1R signaling regulates macrophage survival, proliferation, differentiation, and metabolism through the RAS/RAF/MEK/ERK and PI3K/Akt/mTOR pathways, whereas TGF-β modulates macrophage function through Smad-dependent and Smad-independent pathways [62,63]. Intracellular pathogens employ diverse strategies to modulate these host signaling pathways, thereby preventing macrophage activation and suppressing its antimicrobial responses [64]. Pathogens such as *Mycobacterium tuberculosis* [65], *Leishmania* spp. [66], *Shigella flexneri*, and *Toxoplasma gondii* [67] interfere with the JAK–STAT and MAPK signaling pathways, disrupting IFN-γ-mediated macrophage activation and impairing the development of an effective antimicrobial phenotype [68]. Similarly, *Leishmania* spp. and *Mycobacterium tuberculosis* attenuate macrophage ROS production by interfering with protein kinase C (PKC)-dependent signaling pathways [69,70]. In addition, several intracellular pathogens, including *Salmonella* spp. [71], *Pseudomonas aeruginosa* [72], *Francisella tularensis* [73], and *Listeria monocytogenes* [74], manipulate the PI3K/Akt signaling pathway to promote anti-inflammatory responses, inhibit apoptosis, and enhance intracellular survival [75]. These alterations facilitate immune evasion and enable persistent infection within macrophages.

#### 2.3.4. Manipulation of Macrophage Polarization

Macrophage polarization is a key determinant of their functional phenotype and antimicrobial activity. Generally, IFN-γ- and PAMP-driven M1-like polarization promotes phagolysosomal maturation and enhances intracellular bacterial killing. In contrast, IL-4-, IL-10-, and tissue repair-associated signals promote an M2-like phenotype characterized by predominantly anti-inflammatory and tissue-reparative functions, which can provide a permissive environment for bacterial persistence and intracellular replication. Intracellular bacteria can manipulate macrophage polarization to promote their survival and persistence within host cells [40]. Most intracellular pathogens, including *Salmonella typhimurium*, *Ehrlichia muris*, *Chlamydia pneumoniae*, *Borrelia burgdorferi*, and *Rickettsia conorii*, suppress M1-like polarization and promote M2-like polarization, thereby creating a permissive intracellular niche that facilitates bacterial persistence. However, exceptions to this classical paradigm have also been reported [76]. Certain intracellular pathogens, such as *Shigella dysenteriae*, *Rickettsia montanensis*, *Listeria monocytogenes*, and *Legionella pneumophila*, induce M1 polarization during specific stages of infection. The resulting pro-inflammatory response promotes the secretion of cytokines and chemokines that recruit additional immune cells, including monocytes and neutrophils, which may subsequently serve as host cells for bacterial infection and dissemination. Furthermore, inflammation-induced tissue damage can disrupt tissue barriers and release nutrients, indirectly supporting bacterial survival and spread [77]. In contrast, pathogens such as *Mycobacterium tuberculosis*, *Mycobacterium leprae*, and *Coxiella burnetii* exhibit stage-dependent modulation of macrophage polarization. During the early phase of infection, these pathogens often induce M1 polarization, whereas chronic infection is characterized by a predominance of M2 macrophages, frequently coexisting with M1 macrophages, thereby facilitating long-term persistence within the host [40,78,79].

#### 2.3.5. Blockade of Phagosome Maturation

Another strategy employed by intracellular pathogens is to prevent or delay phagosome maturation and subsequent phagolysosome formation, thereby promoting bacterial survival within macrophages. Phosphatidylinositol 3-phosphate (PI3P) enrichment and the Rab5-to-Rab7 transition are key regulatory events in phagosome maturation, facilitating the progression from early to late phagosomes and their subsequent fusion with lysosomes [80,81]. Intracellular pathogens such as Mycobacterium tuberculosis and Salmonella enterica arrest phagosome maturation through the depletion of phosphatidylinositol 3-phosphate (PI3P), and interference with Rab GTPase-mediated vesicular trafficking [82,83]. *Legionella pneumophila* utilizes its type IV secretion system (Dot/Icm) to translocate numerous effector proteins into the host cytosol, leading to the formation of an endoplasmic reticulum-derived replicative vacuole that avoids lysosomal fusion [84]. In *Leishmania* spp., the surface glycoconjugate lipophosphoglycan (LPG) interferes with phagosome maturation and delays phagosome–lysosome fusion [85]. Unlike these pathogens, *Coxiella burnetii* does not permanently block phagolysosome formation. Instead, its type IV secretion system remodels the phagosomal compartment during maturation, allowing the bacterium to survive and replicate within a specialized acidic phagolysosome-like vacuole known as the Coxiella-containing vacuole (CCV) [86].

#### 2.3.6. Evasion of Free Radical Mediated Bacterial Killing

Following pathogen engulfment, macrophages initiate a phagocytic oxidative burst that generates reactive oxygen species (ROS), which contribute to intracellular pathogen killing [87]. However, many intracellular pathogens have evolved strategies to evade ROS-mediated bactericidal activity, either by inhibiting ROS production through interference with NADPH oxidase assembly or by detoxifying the ROS generated within the phagosome [36]. For example, *Salmonella enterica* employs type III secretion system (T3SS) effectors to disrupt the assembly of the NADPH oxidase complex on the phagosomal membrane, thereby limiting ROS production [88]. Similarly, the surface lipophosphoglycan (LPG) of *Leishmania* inhibits NADPH oxidase assembly and also functions as a free-radical scavenger, protecting the parasite from oxidative damage [89]. In addition, several intracellular pathogens, including *Salmonella*, *Yersinia* spp., and *Listeria monocytogenes* and *Pseudomonas aeruginosa*, express antioxidant enzymes such as superoxide dismutase, catalase, and peroxidases that neutralize ROS and promote intracellular survival. Inducible nitric oxide synthase (iNOS)-mediated production of reactive nitrogen intermediates (RNIs) represents another critical antimicrobial mechanism of activated macrophages. To counter this defense, pathogenic *mycobacteria* modulate host arginine metabolism, including the induction of arginase-1, which competes with iNOS for L-arginine and consequently reduces nitric oxide production. Furthermore, pathogens such as *Salmonella* and *Mycobacterium* spp. employ additional effector-mediated mechanisms to detoxify or evade the antimicrobial effects of RNIs, thereby enhancing their persistence within macrophages [90,91].

#### 2.3.7. Manipulation of Nutrients and Metabolic Environment

Intracellular bacteria employ diverse nutrient acquisition strategies to overcome host nutritional restrictions and obtain essential metabolites required for their survival and replication [92]. *Salmonella enterica* remodels the endosomal system through the formation of Salmonella-induced filaments (SIFs), facilitating access to host-derived nutrients, including amino acids such as alanine, aspartate, proline, and glutamate [93]. Similarly, intracellular pathogens such as *Chlamydia trachomatis*, *Legionella pneumophila*, and *Coxiella burnetii* manipulate host membrane contact sites to promote non-vesicular lipid transport and acquire host lipids required for membrane biogenesis and intracellular growth [94]. Iron acquisition represents another critical determinant of intracellular survival. To overcome host nutritional immunity, many intracellular pathogens secrete high-affinity iron-chelating molecules known as siderophores, which scavenge iron from host iron-binding proteins, including transferrin and lactoferrin [95]. Furthermore, intracellular pathogens exploit host metabolic pathways as carbon and energy sources. For example, *Salmonella* utilizes host glycolytic metabolites to support intracellular replication, whereas *Mycobacterium tuberculosis* preferentially exploits host fatty acids and cholesterol as major carbon sources during infection. These metabolic adaptations enable intracellular pathogens to thrive within the nutrient-limited environment of host cells [96].

#### 2.3.8. Autophagy Evasion

Autophagy is a fundamental cellular process responsible for the degradation and recycling of cytosolic components and damaged organelles. During this process, the cargo is sequestered within a double-membrane vesicle known as an autophagosome, which subsequently fuses with lysosomes to form an autolysosome, where the cargo is degraded [97]. In addition to its homeostatic functions, autophagy serves as an important host defense mechanism against intracellular pathogens through a selective process known as xenophagy [98,99]. However, many intracellular pathogens have evolved diverse strategies to evade or subvert autophagic clearance [100]. For example, *Shigella flexneri* masks the surface protein IcsA (VirG) to avoid recognition by the autophagy machinery [101]. *Listeria monocytogenes* expresses Internalin K (InlK), which recruits major vault protein (MVP) to camouflage the bacterium from autophagic detection [102]. *Mycobacterium tuberculosis* suppresses autophagy by inducing host microRNAs that target key components of the autophagy pathway [103]. Similarly, *Streptococcus pyogenes* utilizes Streptolysin O, a pore-forming cytotoxin, to damage the autophagosomal membrane and disrupt the autophagic flux [104]. *Salmonella enterica* utilizes the T3SS effector, SseL, to remove ubiquitin chains from the Salmonella-containing vacuole, thereby impairing ubiquitin-mediated recognition and autophagic clearance of intracellular bacteria [105]. Importantly, Abhinand et al. reported an epigenetic mechanism through which intracellular *Klebsiella pneumoniae* modulates macrophage immune responses. Intracellular *Klebsiella pneumoniae* infection upregulates histone deacetylase 2 (HDAC2), resulting in deacetylation of H3K18. This alteration is associated with reduced autophagic activity, increased IL-10 production, and an M2-like, immunosuppressive macrophage phenotype [106].

Collectively, intracellular pathogens employ diverse strategies to subvert macrophage immune surveillance, thereby establishing a permissive intracellular niche for their persistence and replication (Figure 2). These multifaceted interactions highlight host–pathogen interaction pathways as potential therapeutic targets and provide a rationale for the development of host-directed therapies (HDTs) as complementary strategies for the treatment of intracellular bacterial infections.

## 3. Host Defense Peptides

Host defense peptides (HDPs) are short polypeptides that constitute an evolutionarily conserved component of the innate immune system and are found across a wide range of organisms, from bacteria to vertebrates. Most HDPs are amphipathic, comprising 10–50 amino acids and possessing a net positive charge at physiological pH. These properties facilitate electrostatic interactions with the negatively charged membranes of microbial cells, leading to membrane disruption and pathogen killing [107]. Owing to their potent broad-spectrum activity against Gram-positive and Gram-negative bacteria, fungi, viruses, and parasites, HDPs were initially recognized as antimicrobial peptides (AMPs). The first widely recognized antimicrobial peptide preparation, tyrothricin, was obtained by René J. Dubos in 1939 from the soil bacterium *Bacillus brevis*. Tyrothricin contained gramicidin D—a mixture dominated by gramicidin A together with gramicidin B and C—and tyrocidine [108,109]. Subsequently, numerous AMPs have been identified from both vertebrate and invertebrate species [110]. According to the Antimicrobial Peptide Database (APD), version APD6, as of January 2026, a total of 6309 peptides have been catalogued, including 3379 naturally occurring peptides, 2290 synthetic peptides, and 373 predicted AMPs (https://aps.unmc.edu/ accessed on 15 July 2026).

Despite their potent antimicrobial activity *in vitro*, the efficacy of many AMPs is significantly reduced under physiological conditions. Their antimicrobial function can be impaired by the presence of divalent cations, anionic polysaccharides, F-actin, glycosaminoglycans, and other components present in body fluids, blood, and mucosal secretions. Consequently, only a limited number of AMPs have successfully progressed through clinical development as direct antimicrobial agents [13]. Interestingly, the immunomodulatory functions of these peptides remain largely preserved under physiological conditions. This observation has shifted research interest from viewing these molecules solely as antimicrobial peptides to recognizing them as host defense peptides, which can modulate innate and adaptive immune responses while promoting a balanced inflammatory reaction. Such properties make HDPs attractive candidates for host-directed therapeutic strategies against infectious diseases [111,112,113].

### 3.1. Classification

Host defense peptides (HDPs) are classified based on their structural conformation, source, amino acid composition, and biological activity (Table 2). According to the structure, HDPs are generally grouped into four major categories: α-helical peptides, β-sheet peptides, linear extended peptides, and mixed α-helical/β-sheet peptides. Cyclic or complex peptide topologies are also reported for many AMPs. α-Helical peptides are the most common class of HDPs. They typically adopt a random coil structure in aqueous solution but undergo a conformational transition to an α-helix upon interaction with microbial membranes. Most α-helical peptides lack cysteine residues and disulfide bonds. β-Sheet peptides are characterized by the presence of cysteine residues that form intramolecular disulfide bonds, which stabilize the peptide structure. These disulfide bonds confer enhanced structural rigidity, making β-sheet peptides more resistant to proteolytic degradation than many other HDP classes. Linear extended peptides do not possess a defined secondary structure and exhibit considerable conformational flexibility in solution and upon membrane interaction. These peptides often exhibit a high abundance of one type of amino acid, typically glycine, proline, and tryptophan [114]. Mixed α-helical/β-sheet peptides contain both α-helical and β-sheet structural elements. As a result, they combine the membrane-interacting flexibility of α-helical peptides with the structural stability associated with β-sheet peptides. In addition to these classes, some HDPs exist as cyclic peptides, characterized by a closed-loop conformation formed through covalent linkage between the peptide termini or specific amino acid residues. The cyclic structure enhances their stability, resistance to proteolytic degradation, and biological activity under diverse physiological conditions [115,116].

HDPs can also be classified based on their amino acid composition, particularly when a specific amino acid constitutes more than 25% of the peptide sequence. Accordingly, they are categorized into proline-rich, glycine-rich, histidine-rich, arginine-rich, and tryptophan-rich peptides. Proline-rich host defense peptides (PrHDPs) exhibit a mechanism of action distinct from that of membrane-disruptive HDPs. Instead of causing membrane damage to enter the bacterial cells, PrHDPs are actively transported across the bacterial inner membrane via specific transporters such as SbmA. Once inside the cytoplasm, they target the ribosome and inhibit protein synthesis by preventing the binding of aminoacyl-tRNA at the peptidyl transferase center [117]. Tryptophan-rich HDPs are characterized by abundant hydrophobic tryptophan residues, which preferentially interact with the interfacial region of the lipid bilayer. These interactions facilitate peptide insertion into the membrane and contribute to membrane destabilization [118]. Arginine-rich HDPs contain multiple positively charged arginine residues that promote strong electrostatic interactions and hydrogen bonding with the negatively charged components of bacterial cell membranes, such as phospholipids and lipopolysaccharides [119]. The complementary physicochemical properties of tryptophan and arginine can confer potent and broad-spectrum antimicrobial activity to Tryptophan- and Arginine-rich HDPs. Tryptophan facilitates membrane insertion through hydrophobic interactions, while arginine enhances membrane binding through electrostatic attraction. The combined contribution of these residues can elicit efficient membrane penetration and potent antimicrobial activity [120,121]. Similarly, Histidine-rich HDPs exhibit strong membrane-permeating properties, while glycine-rich HDPs typically contain 14–22% glycine residues, which significantly influence their secondary structure. Glycine-rich peptides are widely distributed in nature and include peptide families such as attacins and diptericins [112,122].

Natural HDPs have been identified across diverse taxa spanning the six kingdoms of life, including Bacteria, Archaea, Protista, Fungi, Plantae, and Animalia. According to the AMP Database (APD6), a total of 3379 natural HDPs have been catalogued based on their biological source. These include 430 from bacteria, 5 peptides from archaea, 8 from protists, 29 from fungi, 272 from plants, and 2628 from animals. Animal-derived HDPs represent the largest and most diverse group and can be further classified according to their taxonomic origin. The APD6 database reports 156 human antimicrobial peptides, 408 biologically active peptides from other mammals, 1113 peptides from amphibians, 163 peptides from fish, 55 from reptiles, 48 from birds, 696 from arthropods, 54 from molluscs, and 6 peptides from protozoa, among others. This diversity and widespread distribution of HDPs in nature underscore their essential role as an innate immune component in distinct organisms (https://aps.unmc.edu/).

HDPs can also be categorized according to their pharmacological activities. The APD6 database classifies HDPs into 25 functional categories based on their reported biological effects. Among these, antibacterial peptides constitute the largest group, accounting for approximately 60% of all reported HDPs, followed by antifungal peptides, which represent nearly 26%. Antiviral, antiparasitic, and antimalarial activities have also been extensively documented, highlighting the therapeutic potential of HDPs against a broad spectrum of infectious diseases. Beyond their antimicrobial properties, HDPs exhibit diverse immunomodulatory and therapeutic functions, including anti-inflammatory, antioxidant, anticancer, antidiabetic, and wound-healing activities. These multifunctional properties have attracted considerable interest in the development of HDP-based therapeutics for both infectious and non-communicable diseases [123].

**Table 2:** Classification of host defense peptides.

Sl. No	Classification	Characteristics	Representative Examples with Sequence Information
1.	**Based on structural characteristics **		
	α-helix [123,124]	• Most studied • Most abundant • Undergo conformational transition from linear structure to an α-helix upon interaction with microbial membranes • No cysteine residues • Abundant hydrophobic regions	• LL-37(LLGDFFRKSKEKIGKEFKRIVQRI KDFLRNLVPRTES) • Magainin 1 (GIGKFLHSAGKFGKAFVGEIMKS) • Pleurocidin (GWGSFFKKAAHVGKHVGKAALTHYL) • Melittin (GIGAVLKVLTTGLPALISWIKRKRQQ) • BM-Moricin (AKIPIKAIKTVGKAVGKGLRAINIA STANDVFNFLKPKKRKA)
	β-sheet [125,126]	• Cysteine-rich • Form disulfide bonds which confer structural stability and resistant to protease-mediated degradation	• Protegrin 1(RGGRLCYCRRRFCVCVGR) • Thanatin (GSKKPVPIIYCNRRTGKCQRM) • Tachyplesin 1 (KWCFRVCYRGICYRRCR) • Polyphemusin (RRWCFRVCYRGFCYRKCR) • Gomesin (QCRRLCYKQRCVTYCRGR)
	αβ-Peptides (Mixed) [125,126]	• Contain both α-helix and β-sheet • Possess flexibility of α-helical peptides with the structural stability associated with β-sheet peptides	• Human β-Defensin-2(GIGDPVTCLKSGAICHPVF CPRRYKQIGTCGLPGTKCCKKP) • Human β-Defensin-3(GIINTLQKYYCRVRGGR CAVLSCLPKEEQIGKCSTRGRKCCRRKK)
	Non-αβ Peptides (Linear or loop extended structures) [114,127]	• No regular secondary structure • Attain extended, loop, turn, or spiral structures without α-helix or β-strands	• Indolicidin (ILPWKWPWWPWRR) • PR-39 (RRRPRPPYLPRPRPPPFFPPRLPPRIP PGFPPRFPPRFP) • KAMP-10 (GGLSSVGGGS)
	Cyclic peptides [128,129]	• Head-to-tail cyclization (N-terminus to C-terminus) or side chain connections • Highly stable • Broad-spectrum antimicrobial activity	• Nisin A (ITSISLCTPGCKTGALMGCNMK TATCHCSIHVSK) • Retrocyclin-1 (GICRCICGRGICRCICGR) • Gramicidin S (VKLFPVKLFP)
2.	**Based on amino acid composition**		
	Proline Rich [130,131]	• Non-polar amino acid • Enter cytoplasm via Sbma transporter • Target ribosome and inhibit protein synthesis	• Bac7(RRIRPRPPRLPRPRPRPLPFPRPGPRPIPR PLPFPRPGPRPIPRPLPFPRPGPRPIPRPL) • Bac5(RFRPPIRRPPIRPPFYPPFRPPIR PPIFPPIRPPFRPPLGPFP) • PR-39(RRRPRPPYLPRPRPPPFFPP RLPPRIPPGFPPRFPPRFP)
	Tryptophan and Arginine Rich [121,132]	• Non-polar amino acid • Penetrate membrane easily • Tryptophan affects lipid bilayer interface • Arginine confers positive charge and hydrogen bond interaction to peptide • Tryptophan-arginine combination shows enhanced membrane penetration	• Indolicidin(ILPWKWPWWPWRR) • Tripticin(VRRFPWWWPFLRR)
	Histidine Rich [133]	• Good membrane permeation • Active under acidic condition	• Histatin5 (DSHAKRHHGYKRKFHEKHHSHRGY) • Histatin 3 DSHAKRHHGYKRKFHEK HHSHRGYRSNYLYDN) • Histatin 8 (KFHEKHHSHRGY)
	Glycine Rich [123,134]	• Flexible structure • Widely exist in nature	• Holotricin3 (YGPGDGHGGGHGGGHGGGH GNGQGGGHGHGPGGGFGGGHGGGH GGGGRGGGGSGGGGSPGHGAGGGYPGGHG GGHHGGYQTHGY) • Armadillidin (GHLGRPYIGGGGGFNRGGGFH RGGGFHRGGGFHSGGGFHRGGGFHSGG SFGYR) • Hymenoptaecin (EFRGSIVIQGTKEGKSRPSL DIDYKQRVYDKNGMTGDAYGGLNIRPGQ PSRQHAGFEFGKEYKNGFIKGQSEVQRG PGGRLSPYFGINGGFRF)
3.	**Based on sources**		
	Mammals [135]	• Largest proportion of characterized AMPs	• Human β-Defensin-2(GIGDPVTCLKSGAICHPVF CPRRYKQIGTCGLPGTKCCKKP) • LL-37(LLGDFFRKSKEKIGKEFKRIVQR IKDFLRNLVPRTES) • Lactoferricin B (FKCRRWQWRMKKLGA PSITCVRRAF)
	Amphibians [136]	• Found in skin secretions of frogs, toads, and salamanders, etc.	• Magainin 1 (GIGKFLHSAGKFGKAFVGEIMKS) • Dermaseptin-B2 (GLWSKIKEVGKEAAKAAAKAAGK AALGAVSEAV) • Temporin A (FLPLIGRVLSGIL)
	Insects [137]	• Mainly synthesized in fat bodies and blood cells of insects	• Cecropin B (KWKVFKKIEKMGRNIRNGIVKAGP AIAVLGEAKAILS) • Thanatin (GSKKPVPIIYCNRRTGKCQRM)
	Fish [138,139]	• Primarily produced in epithelial tissues (skin, gills, intestine) and immune cells	• Chionodracine (FFGHLYRGITSVVKHVHGLLSG) • Piscidin (KGARQAWKDYKYNRNMQKMNQGYGQQGG) • aCATH (RMRRSKSGKGSGGSKGS GSKGSKGSKGSGSKGSGSKGGSRPGG GSSIAGGGSKGKGGTQTA)
	Plants [140,141]	• Extracted and isolated from the stems, seeds, and leaves of various plants	• Gamma1-purothionin (KICRRRSAGFKGPCMSNKNCAQVCQE QWQQQNCDQPFRRCKCIRQC) • Snakin-1(GSNFCDSKCKLRCSKAGLADRC LKYCGICCEECKCVPSGTYGNKHECPCYR DKKNSKGKSKCP) • Circulin A (GIPCGESCVWIPCISAALGCSCKN KVCYRN)
	Marine organisms [142,143]	• Diverse owing to the absence of adaptive immunity in most marine invertebrates Often rich in cysteine residues and stabilized by multiple disulfide bonds, conferring high stability	• Tachyplesin I (KWCFRVCYRGICYRRCR) • Clavanin A (VFQFLGKIIHHVGNFVHGFSHVF) • Styelin A(GFGKAFHSVSNFAKKHKTA) • Penaeidin-1 (YRGGYTGPIPRPPPIGRPPLRLVV CACYRLSVSDARNCCIKFGSCCHLVK)
	Microorganisms [144,145]	• Obtained from microorganisms like bacteria and fungi	• Nisin Z (ITSISLCTPGCKTGALMGCNMKTAT CNCSIHVSK) • Subtilin (WKSESLCTPGCVTGALQTCFLQTLTCN CKISK) • Mersacidin (CTFTLPGGGGVCTLTSECIC) • Geobacillin 1(VTSKSLCTPGCITGVLMCLTQNSC VSCNSCIRC)

Sequence information was obtained from the Antimicrobial Peptide Database (APD6). Additional information, including the original reference, can be found in the APD [146].

### 3.2. Host Defense Peptides as Immunomodulators Against Intracellular Bacteria

Growing evidence highlights the host defense peptides (HDPs) as effective therapeutic interventions for intracellular bacterial infections via modulation of host antimicrobial immunity [147]. Intracellular pathogens employ diverse mechanisms to alter host cell physiology and evade immune surveillance, as described in Section 2. In this section, we discuss the therapeutic potential of HDPs against various intracellular bacterial pathogens and the host-directed mechanisms through which they exert their antimicrobial effects (Figure 3).

#### 3.2.1. *Mycobacterium tuberculosis*

*Mycobacterium tuberculosis* is an acid-fast bacillus and a highly adaptable human pathogen. It employs diverse strategies to evade and manipulate host immune responses, facilitating intracellular persistence in macrophages and the establishment of tuberculosis. Its ability to survive within alveolar macrophages presents a major challenge for effective antimicrobial therapy. In this context, HDPs have considerable therapeutic potential, as they target intracellular *M. tuberculosis* via modulation of host immune responses [148].

Among the HDPs, LL-37, a member of the cathelicidin family, is one of the most extensively studied peptides against *Mycobacterium tuberculosis.* LL-37 enhances the microbicidal activity of macrophages by promoting the fusion of mycobacteria-containing phagosomes with lysosomes, thereby facilitating bacterial clearance [149,150]. Torres-Juarez et al. reported LL-37 as an immunomodulatory AMP in infected macrophages. They showed that exogenous LL-37 treatment decreased the production of TNF-α and IL-17 while inducing TGF-β and IL-10 in mycobacteria-infected macrophages [151]. The direct antimicrobial activity of LL-37 may be beneficial during the early stages of infection. However, as the disease progresses, the immunomodulatory effects of LL-37 may help control hyperinflammatory responses, prevent pathogen dissemination, and preserve pulmonary function [152]. Importantly, Mily et al. have shown that phenylbutyrate induces the expression of the host defense peptide LL-37 in humans, which enhances vitamin D-dependent autophagy and facilitates the clearance of intracellular *Mycobacterium tuberculosis* [153].

Human neutrophil peptide-1 (HNP-1) is a well-characterized antimicrobial peptide belonging to the α-defensin family. Studies have shown that HNP-1 binds to *Mycobacterium tuberculosis*-infected macrophages and enters the cytosol. Within macrophages, HNP-1 exerts potent antimycobacterial activity by inhibiting bacterial DNA and lipid biosynthesis. Importantly, HNP-1 exhibits low toxicity toward host macrophages, highlighting its potential as a safe and promising candidate for tuberculosis therapy [154]. Its efficacy was further validated in a murine model of experimental tuberculosis, where treatment with HNP-1 significantly reduced the bacterial burden in the lungs, liver, kidneys, and spleen [155].

Hepcidin is a host defense peptide that plays a central role in iron homeostasis by regulating the iron exporter ferroportin. Park et al. demonstrated that co-culture of bacterioferritin A-treated macrophages with T cells induced hepcidin expression, leading to enhanced killing of intracellular *Mycobacterium tuberculosis* [156]. In another study, hepcidin was shown to localize to infected phagosomes, where it exerted direct antimycobacterial activity. These findings suggest that, in addition to its role in iron regulation, hepcidin contributes to host defense against intracellular *Mycobacterium tuberculosis* infection [157].

Lactoferrin is an iron-binding glycoprotein belonging to the transferrin family. It is present in most exocrine secretions, including milk, tears, saliva, and respiratory and intestinal secretions. Recent studies have implicated lactoferrin as a host defense peptide against intracellular *Mycobacterium tuberculosis* [158]. Lactoferrin treatment effectively kills intracellular *Mycobacterium tuberculosis* through multiple mechanisms, including IFN-γ-mediated nitric oxide (NO) synthesis [159], increased phagocytic activity, stimulation of lysosome and autophagosome formation, and enhanced phagosome maturation [160,161]. Lactoferrin also activates adaptive immunity by increasing the IL-12:IL-10 ratio and promoting TH1 cell responses, MHC class II expression, CD40 expression, and T-cell stimulation [162]. Importantly, lactoferrin has been reported to balance the immunopathology associated with TB infection, which can lead to pulmonary dysfunction, by suppressing pro-inflammatory cytokines such as TNF-α, IL-6, and IL-1β [163]. Similarly, innate defense regulator (IDR) peptides, such as IDR-1002, IDR-HH2, and IDR-1018, have also been reported to function as host-directed therapeutics against intracellular *Mycobacterium tuberculosis*. These peptides inhibit *Mycobacterium tuberculosis* persistence within macrophages by modulating macrophage polarization toward an intermediate M1–M2 phenotype, inducing chemokine production, enhancing the recruitment of phagocytic and adaptive immune cells, and suppressing lung inflammation [164,165].

#### 3.2.2. *Salmonella enterica*

*Salmonella enterica* is a Gram-negative intracellular pathogen that is classified into typhoidal and non-typhoidal serovars. The typhoidal serovars, *Salmonella enterica* serovar Typhi and serovar Paratyphi, are the causative agents of typhoid and paratyphoid fever, respectively. In contrast, the non-typhoidal serovars, including *Salmonella enterica* serovar Enteritidis and serovar Typhimurium, primarily cause intestinal inflammation and diarrhea [28].

HDPs have been investigated as therapeutic agents against intracellular *Salmonella* infections owing to their ability to modulate the immunosuppressed host microenvironment in addition to their direct antimicrobial activity. Maiti et al. evaluated the combinatorial effect of bacteria-derived recombinant human β-defensins (HBD-1 and HBD-2). Treatment with a 1:1 combination of HBD-1 and HBD-2 significantly improved the survival of *Salmonella*-infected mice while reducing tissue damage and sepsis [166]. Similarly, another team demonstrated a synergistic combination of plant-derived recombinant β-defensin for the management of salmonellosis. These defensins exert direct microbicidal effects through endotoxin neutralization and enhance bacterial clearance by promoting CCR6-mediated immune cell recruitment and bridging innate and adaptive immune responses [167]. Importantly, the antimicrobial peptide AP2 exhibited a protective effect against *Salmonella Typhimurium* infection by remodeling the gut microbiota and regulating immune responses, thereby enhancing host defense mechanisms against bacterial infection [168]. Similarly, Wang et al. investigated the effects of the antimicrobial peptide JH-3 in *Salmonella Typhimurium*-infected macrophages. Their results demonstrated that JH-3 significantly reduced the intracellular survival of *S. Typhimurium*, attenuated macrophage damage, and suppressed *Salmonella*-induced production of the pro-inflammatory cytokines IL-2, IL-6, and TNF-α [169].

#### 3.2.3. *Brucella*

*Brucella* is an aerobic, Gram-negative bacterium and a facultative intracellular pathogen. The pathogenicity of *Brucella* primarily depends on its ability to evade host immune defense and persist within host cells, particularly macrophages, dendritic cells, and placental trophoblasts. Several AMPs have been reported to function as host-directed immunomodulators against *Brucella* infection [170].

Arayan et al. evaluated the antimicrobial peptide HPA3P against *Brucella abortus* 544. The study demonstrated that HPA3P did not exhibit direct microbicidal activity; instead, it inhibited bacterial internalization and intracellular survival within macrophages. The reduction in intracellular survival of *Brucella abortus* was associated with the modulation of key cytokines, including TNF-α and IFN-γ [171]. Similarly, another group synthesized 20 de novo amphipathic peptides as analogues of naturally occurring peptides. These compounds did not display direct antimicrobial activity against *B. abortus*. However, treatment of chronically infected BALB/c mice resulted in a significant reduction in bacterial burden. The peptides preferentially lysed infected macrophages, thereby exposing intracellular *Brucella* to host immune responses and facilitating bacterial clearance [172]. Zhang et al. synthesized a peptide containing both T-cell and B-cell epitopes derived from OMP31, an outer membrane protein of *Brucella melitensis* that plays a crucial role in bacterial virulence. Peptide immunization induced IgG1 and IgG2 antibody responses and increased the number of IFN-γ-producing T cells in the respiratory draining lymph nodes and spleen. Importantly, *in vivo* administration of the peptide significantly reduced pulmonary bacterial colonization in mice intranasally infected with *B. melitensis* [173]. Additionally, a synthetic peptide targeting OMP25 significantly inhibited the internalization and intracellular survival of *B. abortus* in macrophages. The peptide also reduced the secretion of pro-inflammatory cytokines, including TNF-α and IL-2, suggesting its potential role in modulating host immune responses during *Brucella* infection [174].

#### 3.2.4. *Staphylococcus aureus*

*Staphylococcus aureus* is a Gram-positive bacterium that forms part of the normal microbiota of the skin and nasopharyngeal cavity. However, it is also an important opportunistic human pathogen capable of causing a wide range of diseases, ranging from superficial skin and soft tissue infections to severe conditions such as pneumonia, sepsis, and endocarditis. Traditionally, *Staphylococcus aureus* has been regarded as an extracellular pathogen. However, emerging evidence has established *S. aureus* as a facultative intracellular bacterium capable of invading, surviving, and replicating within various host cells, including macrophages. This intracellular lifestyle contributes to immune evasion, persistence, and recurrent infections [35].

Cecotto et al. evaluated three well-characterized HDPs—human LL-37 (cathelicidin), chicken CATH-2 (cathelicidin), and bovine IDR-1018 (innate defense regulator)—against *Staphylococcus aureus*. Among the three peptides, only CATH-2 exhibited direct antibacterial activity against *Staphylococcus aureus*. Nevertheless, all three peptides demonstrated anti-inflammatory properties by suppressing LPS-induced production of pro-inflammatory cytokines. In the context of intracellular infection, IDR-1018 significantly reduced the number of infected macrophages and decreased the intracellular bacterial burden. Importantly, synergistic combinations of HDPs, either with other HDPs or with conventional antibiotics, have been proposed as an effective strategy to enhance therapeutic efficacy against intracellular *Staphylococcus aureus* infections [175]. Another study demonstrated the potent activity of LL-37 against both extracellular and intracellular *Staphylococcus aureus.* LL-37 exhibited approximately 4000-fold greater antibacterial activity than conventional antibiotics against *Staphylococcus aureus*, including bacteria internalized within osteoblasts [176]. Similarly, two synthetic short peptides, WR-12 and D-IK8, have been reported to possess potent activity against drug-resistant and intracellular *Staphylococcus aureus.* In addition to their antibacterial effects, both peptides demonstrated significant anti-biofilm activity against *Staphylococcus aureus* and *Staphylococcus epidermidis*. In a murine model of methicillin-resistant *Staphylococcus aureus* (MRSA) skin infection, treatment with WR-12 and D-IK8 significantly reduced bacterial burden and attenuated the production of pro-inflammatory cytokines [177]. The synthetic cationic peptide, IDR-1002, demonstrated protective efficacy in a *Staphylococcus aureus* invasive infection model. IDR-1002 induced chemokine production and enhanced recruitment of leukocytes to the infection site [164]. Other HDPs, including Hylin and D1018M, have also shown efficacy against intracellular *Staphylococcus aureus* infections by inhibiting bacterial invasion and modulating the associated inflammatory cytokine responses [178,179]. These findings collectively highlight the potential of HDPs as multifunctional therapeutic agents capable of directly targeting intracellular pathogens while simultaneously regulating host immune responses.

#### 3.2.5. Other Intracellular Bacteria

*Coxiella burnetii* is an obligate intracellular pathogen that primarily replicates within alveolar macrophages. Xiong et al. designed and synthesized novel peptides containing CD4^+^ T-cell epitopes derived from major immunodominant proteins of *Coxiella burnetii* using bioinformatic prediction tools. These peptides stimulated CD4^+^ T-cell proliferation and subsequently induced a Th1-type immune response in mice immunized with *Coxiella burnetii* antigens [180].

*Pseudomonas aeruginosa* can survive intracellularly within host cells, particularly macrophages and epithelial cells, by evading phagosomal killing, modulating host immune responses, and adapting its metabolism to the intracellular environment. Human β-defensins 2 and 3 (HBD2 and HBD3) have been reported to inhibit the intracellular survival of *Pseudomonas aeruginosa* in macrophages. Both peptides enhanced the phagocytosis and intracellular killing of *P. aeruginosa* by downregulating the expression of early growth response genes and the proto-oncogene *c-Fos* [181].

*Listeria monocytogenes* is a facultative intracellular pathogen that invades host cells, escapes from the phagosome, and replicates within the host cell cytosol. Jelleine-1 exhibited potent bactericidal activity against *Listeria monocytogenes*, with a minimum inhibitory concentration (MIC) of 12.5 μg/mL. The peptide increased bacterial membrane permeability, leading to membrane disruption and calcium leakage. In addition to its direct antibacterial effects, Jelleine-1 modulated the host immune response by increasing hemocyte counts, upregulating antimicrobial peptide genes such as *gloverin* and *cecropin D*, and suppressing the production of pro-inflammatory cytokines [182].

Importantly, Kakkerla et al. designed and characterized six antimicrobial peptides against a range of bacterial pathogens, including *Escherichia coli*, *Klebsiella pneumoniae*, *Staphylococcus aureus*, and *Pseudomonas aeruginosa*. Among these, SK1260 exhibited the most potent activity, exerting direct bactericidal effects while simultaneously modulating the host immune response. Specifically, SK1260 reduced the secretion of the pro-inflammatory cytokines IL-6 and TNF-α while maintaining stable levels of the anti-inflammatory cytokine IL-10 in infected macrophages [183].

## 4. Limitations and Translational Challenges of HDP Therapy

Despite a plethora of evidence supporting HDPs as promising therapeutic candidates for the treatment of infectious diseases, only a small fraction has advanced to clinical evaluation (Table 3), underscoring the considerable translational hurdles associated with their development. To date, seven peptide-derived antimicrobials have been approved for clinical use as alternatives to conventional antibiotics. These agents primarily exert direct bactericidal activity through disruption of microbial membranes. Among the approved peptides, vancomycin and daptomycin are the only agents reported to possess experimentally validated immunomodulatory properties in addition to their antimicrobial effects. Studies have shown that these peptides can modulate innate immune responses by enhancing macrophage phagocytic activity, reducing the production of the pro-inflammatory cytokine TNF-α, and regulating the recruitment of innate immune cells in murine models of LPS-induced sepsis. Such findings highlight the therapeutic potential of HDPs beyond direct pathogen killing and support their development as immunomodulatory agents capable of augmenting host defense mechanisms [184].

The limited clinical translation of HDPs is further evident from the DRAMP 4.0 database, which currently contains 30,260 peptide entries, whereas only 96 candidates have progressed to clinical trials [185]. Notably, the clinical development of immunomodulatory HDPs remains limited, with few candidates entering clinical trials and none specifically evaluated for the treatment of intracellular bacterial infections. Several factors have contributed to this narrow development pipeline, including structural complexity, potential host toxicity, unfavorable pharmacokinetic properties, susceptibility to proteolytic degradation in both host and microbial environments, and the often-poor correlation between *in vitro* efficacy and *in vivo* therapeutic outcomes [186].

High manufacturing costs represent a major barrier to the clinical translation of HDPs. Peptide production by solid-phase peptide synthesis (SPPS) is considerably more expensive than the manufacturing of conventional antibiotics, with production costs typically ranging from US$50–400 per gram of peptide, compared with approximately US$0.85 per gram for antibiotics such as aminoglycosides [187,188]. In addition to the economic burden, chemical synthesis of peptides has a substantial environmental impact due to the extensive use of toxic reagents and large volumes of organic solvents. These limitations highlight the need for the development of cost-effective, scalable, and environmentally sustainable manufacturing strategies to support the commercial production of HDPs [110].

Several HDPs have demonstrated potent antimicrobial activity under *in vitro* conditions; however, their efficacy is often markedly reduced under physiological salt and serum conditions. The microbicidal activity of human cathelicidins, including LL-37, and β-defensins is known to be attenuated in the presence of divalent cations, anionic polysaccharides, apolipoproteins, and glycosaminoglycans. These factors interfere with the electrostatic interactions required for peptide binding to bacterial membranes and may compromise the structural stability necessary for antimicrobial activity. In contrast, the immunomodulatory functions of HDPs are generally preserved under physiological conditions, as these effects are largely mediated through interactions with host cell receptors and downstream signaling pathways rather than direct microbial membrane disruption [15,189].

Another major limitation of peptide-based therapeutics is their potential toxicity toward host cells. Owing to their membrane-active nature, many HDPs exhibit limited selectivity between microbial and eukaryotic membranes, resulting in dose-dependent cytotoxicity. Clinically approved peptide antimicrobials such as gramicidin D (mixture of three closely related isoforms–gramicidin A, B, and C) and polymyxins are associated with significant systemic toxicities, including nephrotoxicity, neurotoxicity, and hemolysis [190]. Consequently, their clinical use is largely restricted to topical applications or reserved for the treatment of multidrug-resistant infections when alternative therapeutic options are limited. Similarly, antimicrobial peptides such as indolicidin, melittin, and protegrins have shown potent antimicrobial activity but induce substantial hemolysis and systemic toxicity, which has hindered their clinical development. Likewise, the Phase III clinical development of murepavadin was discontinued due to acute nephrotoxicity. Even LL-37, one of the most extensively investigated HDPs, exhibits hemolytic and cytotoxic effects at high concentrations, highlighting the challenge of balancing antimicrobial efficacy with host–cell safety. These limitations highlight the need for targeted delivery approaches to improve peptide bioavailability, therapeutic efficacy, and safety profiles [191].

Unfavorable pharmacokinetic properties are another major factor limiting the clinical development of HDPs. Friulimicin shares similar physicochemical properties and mechanisms of action with the approved peptide antibiotic, daptomycin. However, its clinical development was discontinued due to differences in its *in vivo* activity, which were attributed to pharmacokinetic limitations. Importantly, detailed pharmacokinetic studies are still lacking for many HDPs, making it difficult to predict their therapeutic performance. Several challenges, including peptide aggregation, rapid clearance from the body, short half-life, and the determination of optimal dosage and dosing frequency, remain to be addressed [15]. Overcoming these limitations will be important for improving the clinical translation of HDPs.

**Table 3:** Peptide-based treatments in clinical trials for bacterial infections.

Peptide and APD ID	Origin	Indication	Mechanism of Action	Clinical Status	Route of Administration	Adverse Effects
Daptomycin (AP02203)	Cyclic lipopeptide from *Streptomyces roseosporus*	Complicated skin and soft tissue infections, *Staphylococcus aureus* bacteremia, right-sided infective endocarditis	Disrupting bacterial membrane potential	Approved	Intravenous	Myopathy, peripheral neuropathy, diarrhoea, hypersensitivity reactions [192]
Colistin (AP02204)	Cyclic polypeptide antibiotic from *Bacillus colistinus*	Binds to LPS and disrupts outer membrane	Last-resort treatment for MDR Gram-negative pathogens	Approved	Intravenous, Inhalational	Nephrotoxicity, neurotoxicity, hypokalemia or hypomagnesemia. [193]
Polymyxin B (AP02928)	Cyclic polypeptide from *Paenibacillus polymyxa*	MDR Gram-negative infections.	Binds to LPS and disrupts outer membrane	Approved	Intravenous, Topical	Nephrotoxicity, hypersensitivity [194]
Gramicidin D (AP0049-gA AP01224-gB AP01225-gC)	Mixture of linear peptide antibiotics (80% gramicidin A, 5% B, and 15% C) from soil bacterium *Bacillus brevis*	Topical use for Superficial skin and ocular infections	Membrane pore formation	Approved	Topical only	Not used systemically due to toxicity (hemolysis, neurotoxicity and other organ toxicity) [195]
Bacitracin (AP04174)	Polypeptide antibiotic produced by *Bacillus licheniformis* and *Bacillus subtilis.*	Topical use for Skin infections caused by Gram-positive bacteria	Inhibits bacterial cell wall synthesis	Approved	Topical-only	Systemic use caused nephrotoxicity and hypersensitivity [196,197] Mild local skin irritation with topical treatment
Pexiganan (AP00952)	Synthetic analogue of magainin	Infected diabetic foot ulcers	Bacterial membrane disruption	Phase III complete; has not received regulatory approval. NCT00563394, NCT00563433	Topical	Mild application-site irritation [198]
Omiganan (AP04794)	Synthetic indolicidin analogue	Catheter-associated infections, Skin antisepsis, Rosacea	Bacterial membrane disruption	Phase III NCT00231153 NCT00608959 NCT02576860	Topical	Mild to moderate application-site erythema, burning, stinging, pruritus, or irritation [199]
hLF1-11 (AP02708)	Human lactoferrin-derived	Bacteremia in immunocompromised patients	Immunomodulatory and antimicrobial activity	Phase II Withdrawn NCT00509938, NCT00430469	Intravenous	Elevation of transaminase
Murepavadin (AP00431)	Synthetic derivative based on the cationic antimicrobial peptide protegrin 1	*Pseudomonas aeruginosa* pneumonia	Targets LptD involved in LPS transport	Phase II Withdrawn NCT03409679	Intravenous	Nephrotoxicity [200]
PL-5 (AP04671)	Synthetic α-helical peptide	Skin and wound infections	Membrane disruption	Phase II NCT06189638	Topical only	Mild application-site reactions [201]
Plectasin (AP00549)	Fungal defensin	Pneumococcal and streptococcal infections	Binds lipid II and inhibits cell wall synthesis	Phase I DRAMP18080	Intravenous	Limited human safety data [202]

Details of the HDPs were also retrieved from the Antimicrobial Peptide Database (APD6), with the corresponding APD accession numbers provided for each peptide. Additional information, including the original reference, can be found in the APD [146].

## 5. Strategies to Improve the Clinical Translation of the Host Defense Peptides

Rational peptide design and chemical modification strategies enhance the proteolytic stability and pharmacokinetic properties of HDPs while reducing their toxicity. Importantly, recent advances in nanotechnology-based delivery systems have enabled efficient intracellular delivery and targeted accumulation of HDPs at infection sites, thereby improving their therapeutic efficacy and safety (Figure 4).

### 5.1. Peptide Engineering and Rational Design of HDP

The therapeutic efficacy of HDPs is influenced by a delicate balance of cationic charge, hydrophobicity, amphipathicity, and structural conformation. At the same time, controlled interactions between HDP amino acid residues and the zwitterionic phospholipids of host cell membranes can improve their safety profile by reducing cytotoxicity. Therefore, rational design of the peptide can contribute to an improved spectrum of activity, safety and stability of peptides [189,203].

Silva et al. employed peptide engineering to develop a synthetic analogue of the HDP mastoparan, a peptide originally isolated from wasp venom, with enhanced antimicrobial and immunomodulatory properties. The authors identified a conserved pentapeptide motif (FLPII) in the N-terminal region of several peptides that was associated with improved antimicrobial and immunomodulatory activity. Incorporation of this motif into the N-terminus of mastoparan significantly enhanced its host defense functions and direct antibacterial activity against *Staphylococcus aureus* and *Escherichia coli*. Importantly, the modified peptide exhibited lower cytotoxicity than the parent peptide [204]. Similarly, another study modified mastoparan using a quantitative structure–activity relationship (QSAR)-based approach, resulting in improved antibacterial activity against Bacillus subtilis. Structural analysis revealed that the modified peptide adopted an α-helical conformation instead of a random coil structure. Furthermore, the presence of tryptophan, lysine, and histidine residues was found to contribute to the enhanced biological activity of the engineered peptide [205]. Another study synthesized different peptides derived from Cathelicidin-BF15 by modifying peptide length, net charge, hydrophobicity, and cyclization through disulfide bond formation. Among the synthesized derivatives, ZY4, a cyclic peptide stabilized by a disulfide linkage, exhibited greater resistance to proteolytic degradation than the parent peptide and demonstrated an *in vivo* half-life of approximately 1.5 h. This engineered peptide showed potent antibacterial activity against both drug-resistant and persister forms of Pseudomonas aeruginosa and *Acinetobacter baumannii*, highlighting the potential of structural optimization strategies to improve peptide stability and therapeutic efficacy [206]. Mohammed et al. synthesized a 12-amino-acid peptide, RR, which exhibited potent antibacterial activity against MRSA. However, its toxicity toward mammalian cells and limited antibacterial spectrum restricted its therapeutic potential. To overcome these limitations, the authors rationally designed and synthesized a series of RR analogues based on several considerations, including maintenance of the α-helical conformation, incorporation of lysine and arginine residues to improve selectivity and reduce toxicity, retention of a peptide length of 12–14 amino acids to minimize toxicity and manufacturing costs, and inclusion of tryptophan, arginine, and lysine residues to enhance antibacterial activity. Among the designed analogues, RR4 demonstrated potent immunomodulatory and antibiofilm activities against multidrug-resistant Pseudomonas aeruginosa and *Acinetobacter baumannii*. Importantly, RR4 exhibited resistance to proteolytic degradation and retained its antibacterial activity in the presence of salts, serum, and acidic pH conditions. Furthermore, the peptide effectively eliminated bacteria residing within macrophages, highlighting its potential for the treatment of intracellular bacterial infections [207,208].

Cyclization is an effective strategy for overcoming the limitations of linear peptides. In their linear form, many HDPs undergo conformational changes that can reduce target binding and biological activity [209]. In addition, linear peptides are more susceptible to proteolytic degradation because their amino acid residues are more accessible to peptidases. To address these limitations, Gunasekera et al. applied peptide engineering approaches, including backbone cyclization and dimerization, to KR-12, an antimicrobial epitope derived from LL-37. Cyclization significantly improved the biological stability and activity of the peptide, while dimerization further enhanced its antimicrobial efficacy [210].

PEGylation is another widely used peptide engineering strategy in which peptides are conjugated with polyethylene glycol (PEG). PEGylation increases the molecular size of peptides, thereby reducing proteolytic degradation through steric hindrance and prolonging systemic circulation by delaying renal clearance. PEGylation and glycosylation of the HDP IDR-1018 were shown to improve its physicochemical properties and immunomodulatory activity. In this study, PEG_6_ was conjugated to the N-terminus of IDR-1018 to generate novel derivatives. The PEGylated analogues exhibited enhanced solubility and reduced peptide aggregation. Moreover, they demonstrated improved safety profiles, with lower toxicity toward mammalian cells and red blood cells than the native peptide. The study also showed that PEGylation preserved the immunomodulatory activity of IDR-1018, whereas glycosylation further enhanced its immunomodulatory effects, as evidenced by increased production of MCP-1 and IL-1RA and reduced levels of TNF-α and IL-1β. Similarly, PEGylated derivatives of the amphibian-derived HDP aurelin also displayed improved safety profiles and a broader antibacterial spectrum compared with the parent peptide [211,212].

Lipidation, achieved through the conjugation of fatty acids to peptides, is another strategy used to improve peptide half-life and bioavailability. Lipidation promotes reversible binding to serum albumin, thereby reducing enzymatic degradation and delaying renal clearance [189]. Incorporation of fatty acids with different chain lengths at the N-terminus of the natural HDP B1 enhanced its microbicidal activity against *Staphylococcus aureus* and *Klebsiella pneumoniae*. Notably, the modified derivatives B1-C6 and B1-C8 exhibited greater biocompatibility and stability than the native peptide [213]. Similarly, anoplin was first N-methylated and subsequently modified by N-terminal fatty acid conjugation to generate N-methylated lipopeptides. These modified derivatives displayed potent antibacterial activity, synergistic interactions with conventional antibiotics, and enhanced resistance to proteolytic degradation [214].

Similarly, the substitution of D-amino acids or other non-canonical amino acids is an effective strategy to improve peptide stability. Such modifications alter both the peptide backbone and side-chain architecture, reducing susceptibility to enzymatic degradation and enhancing resistance to proteases. Replacement of natural L-amino acids with D-amino acids or unnatural amino acids, including D-lysine, D-arginine, L-2,4-diaminobutanoic acid (Dab), L-2,3-diaminopropionic acid (Dap), homoarginine (Hor), α-aminoisobutyric acid (Aib), and thienylalanine, has been shown to improve peptide stability and protease resistance. Among these modified peptides, DP06, which incorporated D-lysine and D-arginine residues, demonstrated excellent stability and a favorable safety profile *in vitro*. However, despite these promising *in vitro* results, DP06 exhibited significant toxicity and reduced biological activity *in vivo.* In contrast, another derivative, UP09, was designed by substituting selected L-lysine residues with Aib or Hor. UP09 showed improved resistance to proteolytic degradation while maintaining good biological activity and an acceptable safety profile in both *in vitro* and *in vivo* studies [215]. These findings highlight the importance of carefully selecting non-canonical amino acid substitutions, as enhanced stability does not always translate into improved *in vivo* performance (Table 4).

### 5.2. Nanotechnology-Based Drug Delivery Systems

Nanotechnology is being increasingly explored to enhance the clinical translation of host defense peptides. Nanoparticle-based delivery systems are employed either to encapsulate HDPs within nanocarriers for passive delivery or to incorporate peptides into surface-modified nanocarriers that enable active targeting to specific sites. Converting HDPs into nano formulations can protect them from enzymatic degradation, improve their bioavailability, and facilitate site-specific delivery. These advantages can enhance therapeutic efficacy while reducing systemic toxicity and off-target effects [216].

#### 5.2.1. Nanoparticle-Based Delivery Systems

Nanoparticle-based delivery systems have emerged as a promising strategy to improve the therapeutic potential of HDPs. These systems can enhance peptide stability, facilitate intracellular delivery, improve bioavailability, and reduce off-target toxicity, thereby addressing several limitations associated with the clinical translation of HDPs.

Gold nanoparticles have been extensively investigated as carriers for HDP delivery. Gold nanoparticles conjugated with a synthetic, non-cytotoxic 16-residue HDP (Au-VGI16KRKP) effectively penetrated macrophages and epithelial cells and exhibited potent bacteriolytic activity against intracellular *Salmonella Typhimurium* while remaining non-toxic to human cells. In a murine Salmonella infection model, the nano formulation demonstrated significant antibacterial efficacy and was found to be pharmacologically safe *in vivo* [217]. Similarly, gold nanoparticle–DNA aptamer conjugates have been employed to deliver histidine-tagged A3-APO peptide into *S. Typhimurium*-infected mammalian cells. This formulation completely inhibited intracellular bacterial colonization both *in vitro* and *in vivo* [218]. Likewise, a gold nanoparticle–DNA aptamer system loaded with histidine-tagged HPA3P peptide showed potent activity against intracellular *Vibrio vulnificus* infections [219].

Silver nanoparticles have also been explored for HDP delivery in antimicrobial applications. Saed et al. developed self-assembled silver–peptide colloidal nanohybrids using a green synthesis approach and demonstrated antibacterial activity against *Escherichia coli* and *Staphylococcus aureus*. The antimicrobial effect was attributed to the combined action of silver-induced ROS generation and peptide-mediated membrane disruption [220]. Interestingly, considering the inherent anti-bacterial action of silver nanoparticles, Thongin et al. combined synthetic HDP, BrSPR20-P1 with silver nanoparticles and reported a synergistic effect of the combination against MRSA and *Escherichia coli* with acceptable safety on erythrocytes and mammalian cells [221].

More recently, a dual drug-loaded dendritic mesoporous silica nanoparticle system (NP@D/AMP) was developed as a molecular sprayer that responds to the acidic microenvironment of infection sites. The formulation encapsulated the HDP G818, which possesses potent antibacterial and antibiofilm properties, along with the small molecule 2-(1,8-naphthyridin-2-yl) phenol (2-NP), a macrophage polarization modulator. Through the combined effects of direct antibacterial activity and host immune modulation, the nano-formulation promoted efficient bacterial clearance. Importantly, NP@D/AMP also regulated the inflammatory microenvironment and enhanced tissue regeneration in an *S. aureus* bone infection model, highlighting its potential as a multifunctional therapeutic platform for the treatment of bacterial infections [222].

#### 5.2.2. Liposomes and Solid Lipid Nanoparticles

Lipid-based nanocarriers, particularly liposomes, have emerged as promising delivery platforms for immunomodulatory peptides for the treatment of intracellular bacterial infections. Liposomal formulations improve the stability, bioavailability, and site-specific delivery of HDPs [223]. Several studies have investigated liposomes encapsulating immunomodulatory peptides either alone or in combination with conventional antibiotics. One such approach involves the development of functional antimicrobial liposomes (FALs), in which the liposome surface is decorated with HDPs linked through a peptide spacer. This design protects the peptides from enzymatic degradation while preserving their antimicrobial activity against *Escherichia coli* and *Staphylococcus aureus*. In addition, FALs demonstrated good biocompatibility, exhibiting minimal toxicity toward mammalian cells [224].

Similarly, Zhang et al. developed a novel 12-amino acid antimicrobial peptide, DP7. Although DP7 exhibited potent antibacterial activity when used in combination with conventional antibiotics in both *in vitro* and *in vivo* studies, high-dose DP7 monotherapy caused severe hemolysis and vascular irritation following intravenous administration. To overcome these limitations, the authors conjugated DP7 with cholesterol, generating an amphiphilic polymer that self-assembled into nano micelles with improved safety and efficacy after systemic administration [225]. Furthermore, liposomes surface-modified with the DP7–cholesterol conjugate and loaded with azithromycin synergistically inhibited MRSA infection through both direct antibacterial activity and activation of the innate immune response, without inducing excessive inflammatory reactions [226]. Another interesting strategy was reported by Hou et al., who developed lipid nanoparticles co-delivering lipidated antimicrobial peptides and mRNA encoding the cathelicidin peptide. The authors first engineered a series of AMP-derived lipids by modifying the TAT peptide (TAT-lipids). Among these, the C6-TAT lipid exhibited superior intracellular penetration and was selected for incorporation into MC3 lipid nanoparticles. This formulation enabled targeted delivery of lipidated peptides to the lungs, resulting in rapid cellular internalization and effective elimination of intracellular bacteria. To simultaneously target extracellular bacteria, the nanoparticles were also loaded with mRNA encoding cathelicidin. Expression and secretion of cathelicidin in the lung tissue provided an additional antimicrobial effect against extracellular pathogens, thereby offering a dual strategy to combat both intracellular and extracellular bacterial infections [227].

#### 5.2.3. Polymeric Nanoparticles

Polymeric nanoparticles have been extensively investigated as carriers for the targeted delivery of HDPs against intracellular bacterial infections. Among these, poly(lactide-co-glycolide) (PLGA) is the most widely studied polymer owing to its biodegradability, biocompatibility, well-defined chemical composition, and ease of controlled manufacturing [228]. PLGA nanoparticles have been successfully employed to deliver an HDP derived from esculentin-1a for the treatment of pulmonary *Pseudomonas aeruginosa* infection [229]. A single intratracheal administration of the peptide-loaded nanoparticles produced approximately a 3-log reduction in bacterial burden that was sustained for up to 36 h. Similarly, Campos et al., encapsulated three HDPs (Dhvar5, MSI-78, and LL-37) into PLGA nanoparticles and compared their release profiles and antibacterial efficacy against *P. aeruginosa*. Dhvar5-loaded nanoparticles exhibited the highest cumulative peptide release (60%) over 72 h, followed by MSI-78-loaded nanoparticles (46%), whereas LL-37-loaded nanoparticles showed only minimal peptide release (6%). Correspondingly, Dhvar5- and MSI-78-loaded nanoparticles demonstrated superior antibacterial activity against *P. aeruginosa*, with MSI-78 nanoparticles exhibiting better cytocompatibility toward mammalian cells [230]. In addition to peptide encapsulation, an alternative strategy involves surface functionalization of PLGA nanoparticles with antimicrobial peptides. For example, grafting MSI-78 onto the surface of PLGA nanoparticles enables peptide exposure on the nanoparticle surface, facilitating direct interaction with bacteria, improving infection-site targeting, and providing a rapid onset of antibacterial activity. Surface-grafted MSI-78 PLGA nanoparticles effectively eradicated *Pseudomonas aeruginosa* and *Staphylococcus aureus* in wound infection models without causing detectable cytotoxicity to mammalian cells [231].

Chitosan nanoparticles have also emerged as promising carriers for HDP delivery because of their biocompatibility, biodegradability, mucoadhesive properties, and inherent antimicrobial activity. Several HDPs, including LL-37, microcin J25, and 3W2, have been encapsulated within chitosan nanoparticles, resulting in enhanced peptide stability, sustained drug release, improved cellular compatibility, and enhanced antibacterial efficacy. In addition, chitosan-based formulations have been reported to preserve or enhance the immunomodulatory and anti-inflammatory activities of encapsulated HDPs, highlighting their potential for the treatment of intracellular bacterial infections [232,233,234].

#### 5.2.4. Macrophage-Targeted Delivery Strategies for Intracellular Bacterial Infections

As most intracellular bacterial pathogens survive and replicate within macrophages by evading or suppressing macrophage-mediated bactericidal mechanisms, macrophage-targeted delivery systems have emerged as a promising strategy for enhancing the therapeutic efficacy of HDPs and other antimicrobial agents against intracellular infections [235].

Chen et al. developed a multifunctional amphiphilic peptide nanoplatform designed for selective macrophage targeting and efficient eradication of intracellular bacteria. The nanoplatform comprises three key functional components: the macrophage-targeting peptide tuftsin (TKPR), the cell-penetrating peptide TAT, and a cathepsin-cleavable Val-Cit linker. The Val-Cit linker was strategically positioned between TKPR and TAT, while the incorporation of six valine residues promoted the self-assembly of the peptide into nanomicelles. Rifampicin was subsequently encapsulated within the hydrophobic core of the nanomicelles (RFP@TVYV). Following binding of the tuftsin moiety to the neuropilin-1 (NP-1) receptor on macrophages, the nanomicelles were internalized through receptor-mediated endocytosis. Inside macrophages, the Val-Cit linker was selectively cleaved by lysosomal cathepsins, exposing the TAT–6-valine fragment and facilitating intracellular release of rifampicin. The released peptide fragment and rifampicin acted synergistically to eliminate intracellular *Staphylococcus aureus* [236].

In another innovative approach, mRNA encoding the antimicrobial peptide 1B376 was engineered with a cathepsin-cleavable linker sequence and encapsulated within vitamin-derived lipid nanoparticles for targeted macrophage delivery. Following cellular uptake, macrophages translated the delivered mRNA into the AMP–cathepsin fusion protein, which subsequently localized to lysosomes. Lysosomal cathepsins cleaved the linker, releasing the active antimicrobial peptide specifically within the intracellular compartment. This strategy enabled efficient killing of multidrug-resistant *Staphylococcus aureus* and *Escherichia coli* residing inside macrophages while minimizing off-target peptide activity [237] (Table 5).

### 5.3. Cell-Penetrating Peptide

Cell-penetrating peptides (CPPs) are short peptides capable of crossing host cell membranes without compromising membrane integrity. Owing to their efficient intracellular uptake, CPPs have been extensively investigated as delivery vehicles for host defense peptides (HDPs) and other therapeutic cargoes for the treatment of intracellular bacterial infections [238].

One such strategy involved AMPN2, a derivative of the marine HDP NZ17074, which was engineered to target intracellular *Salmonella Typhimurium* through covalent conjugation with two CPPs, N2 and Tat II. These CPPs facilitate uptake into infected macrophages primarily via energy-dependent macropinocytosis and clathrin-mediated endocytosis, enabling efficient intracellular delivery of the HDP to eradicate internalized bacteria [239]. Similarly, the chimeric peptide TAT-KR-12 was generated by conjugating the HDP KR-12 with the CPP motif TAT. TAT-KR-12 exhibited potent activity against both planktonic and intracellular *Staphylococcus aureus* while also exerting significant anti-inflammatory effects [240].

Recent studies have further explored nanotechnology-based delivery systems that integrate CPPs with HDPs to enhance targeted intracellular delivery while minimizing off-target toxicity. Zhu et al. developed a self-assembling nano peptide that combines cell-penetrating and antibacterial functions. Functional domains derived from the lead peptides F3FT and N3FT were conjugated with a CPP transactivator peptide and hydrophobic residues to promote self-assembly into nanostructures. The resulting nano peptides efficiently penetrated host cell membranes eliminated intracellular bacteria, and simultaneously suppressed the associated inflammatory response, highlighting their potential as multifunctional therapeutics for intracellular bacterial infections [241].

In another study, a multifunctional chimeric peptide nano assembly comprising four functional domains—a self-assembly core, a cell-penetrating motif, an enzyme-responsive linker, and a targeting ligand—was developed to improve intracellular antibacterial therapy. Under physiological conditions, the peptide self-assembled into nanoparticles. Subsequent ligand–receptor interactions promoted binding to the host cell membrane and triggered a morphological transformation into nanofibers. Bacterial enzyme-mediated cleavage of the enzyme-responsive linker facilitated enhanced cellular uptake and promoted targeted delivery of the therapeutic peptide to intracellular bacteria, thereby improving antibacterial efficacy [242].

In addition to serving as delivery vehicles, few natural or engineered CPPs possess intrinsic antimicrobial activity, making them promising therapeutic candidates against both obligate and facultative intracellular pathogens. These peptides penetrate host cell membranes and eliminate bacteria residing either within the host cytosol or inside membrane-bound vacuoles [243] (Table 6).

## 6. Conclusions and Outlook

In the era of antimicrobial resistance, intracellular bacterial infections represent a major therapeutic challenge, as conventional antibiotics often exhibit limited intracellular penetration and contribute to the emergence of resistant forms. By residing within host cells, intracellular pathogens evade immune surveillance, establish persistent infections, and reduce the efficacy of many existing antimicrobial therapies. In this context, host defense peptides (HDPs) have emerged as promising therapeutic candidates owing to their dual antimicrobial and immunomodulatory functions. Accumulating evidence demonstrates that naturally occurring, synthetic, and engineered HDPs can effectively eliminate intracellular bacteria while restoring host immune function and mitigating the excessive inflammatory responses associated with infection.

Despite these promising findings, the clinical translation of HDPs remains limited by several challenges, including poor intracellular bioavailability, rapid proteolytic degradation, suboptimal pharmacokinetic properties, potential cytotoxicity at higher concentrations, and the high cost of peptide production. Recent advances in peptide engineering and nanotechnology have addressed many of these limitations by improving peptide stability, enhancing targeted intracellular delivery, reducing off-target toxicity, and increasing therapeutic efficacy. Nevertheless, most of these approaches remain at the preclinical stage. Although numerous studies have demonstrated encouraging results in both *in vitro* and animal models, clinical studies evaluating nanocarrier-based HDP formulations for intracellular bacterial infections are still lacking. Future research should focus on establishing standardized preclinical evaluation frameworks, developing clinically relevant intracellular infection models, performing comprehensive immunological and toxicological assessments, and improving scalable and cost-effective manufacturing processes. The integration of artificial intelligence and machine learning with rational peptide engineering offers additional opportunities to accelerate the discovery and optimization of HDPs with improved antimicrobial activity, intracellular targeting, pharmacokinetic properties, and safety profiles.

Overall, the successful clinical translation of HDP-based therapeutics will require coordinated efforts in peptide design, targeted delivery, formulation development, rigorous preclinical and clinical evaluation, scalable manufacturing, and regulatory standardization. Furthermore, advances in precision medicine and personalized therapeutic approaches may facilitate the development of tailored HDP-based interventions for specific pathogens and patient populations.

## Figures and Tables

**Figure 1:**
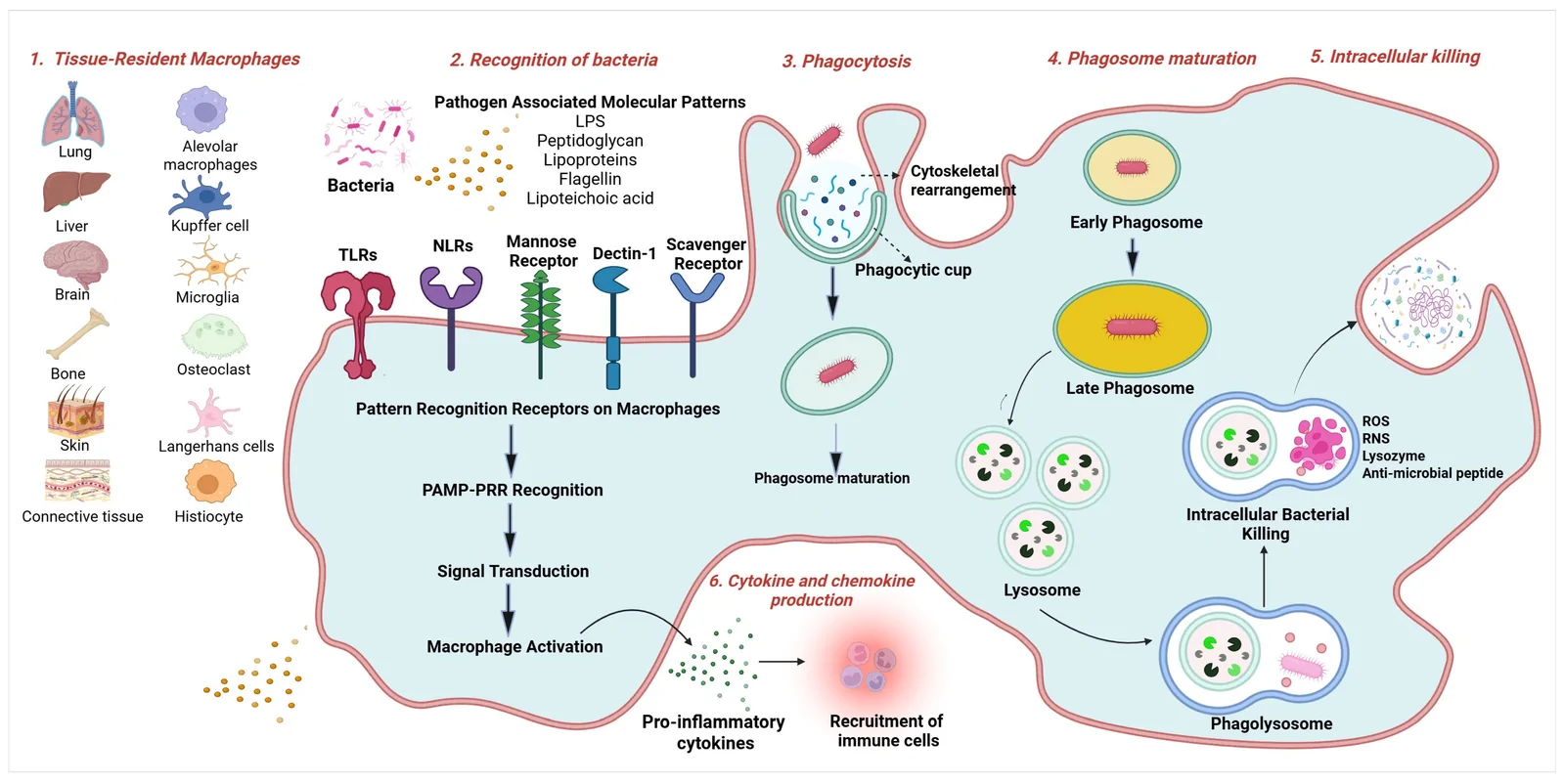
**Innate immune functions of macrophages during bacterial infection:** Macrophages are professional phagocytes that constitute a major component of the innate immune system. They recognize invading bacteria through the interaction between pattern recognition receptors (PRRs) expressed on the macrophage surface and pathogen-associated molecular patterns (PAMPs) present on bacterial cells. This interaction triggers cytoskeletal rearrangement and the formation of a phagocytic cup, which engulfs the pathogen into an intracellular vesicle known as a phagosome. The phagosome subsequently undergoes maturation and fuses with lysosomes to form a phagolysosome. Within this acidic compartment, engulfed bacteria are eliminated by the coordinated action of reactive oxygen species (ROS), nitric oxide (NO), hydrolytic enzymes, and antimicrobial peptides. In addition to their microbicidal activity, activated macrophages secrete pro-inflammatory cytokines and chemokines that recruit and activate other immune cells. They also process and present bacterial antigens via major histocompatibility complex class II (MHC-II) molecules to CD4^+^ T cells, thereby linking innate and adaptive immunity.

**Figure 2:**
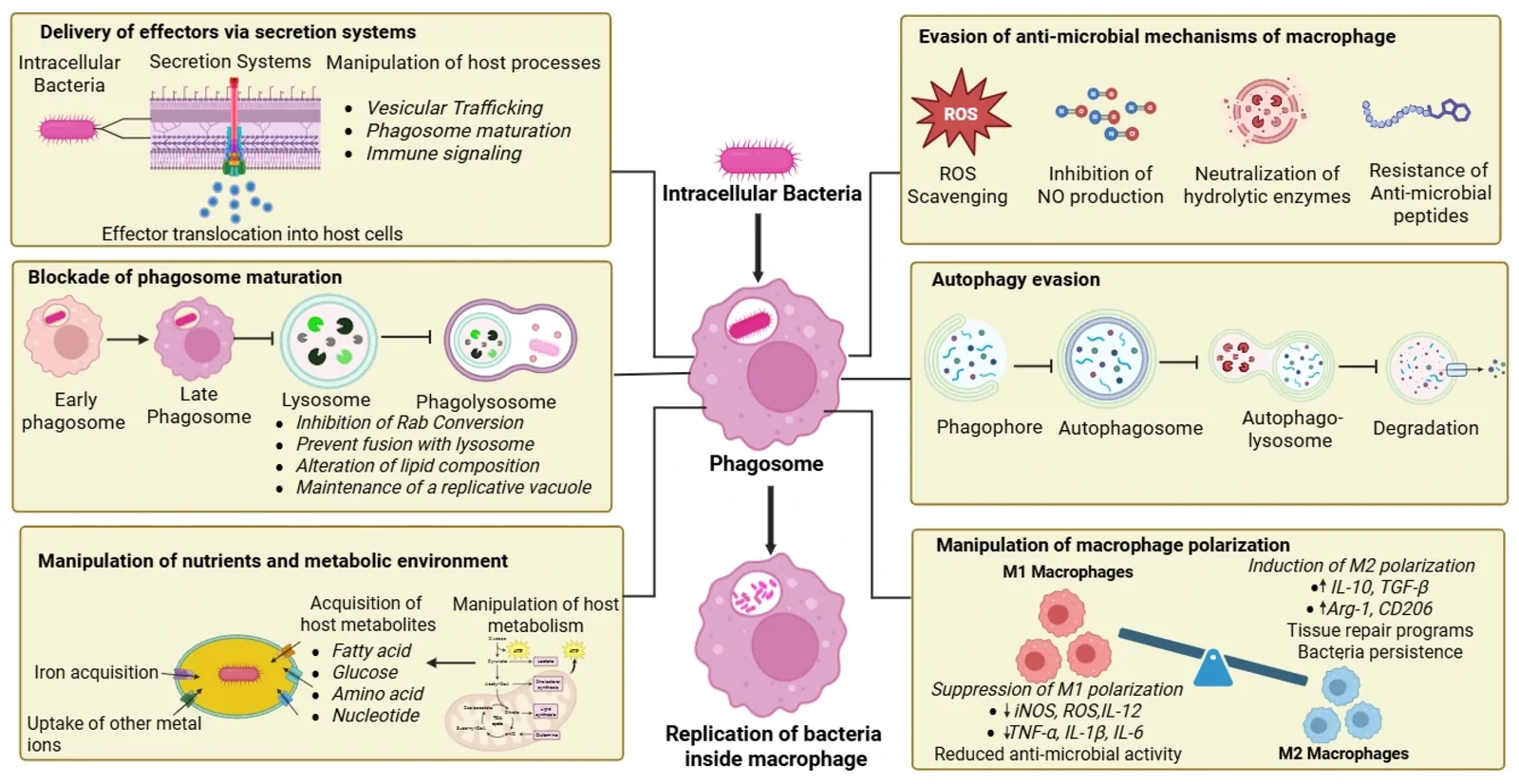
**Mechanisms employed by intracellular bacteria to evade macrophage-mediated killing:** Intracellular bacteria employ multiple strategies to evade the antimicrobial defense mechanisms of macrophages and establish a permissive intracellular niche for survival and replication. These pathogens possess diverse secretion systems that translocate effector proteins into host cells to manipulate host cellular processes and promote intracellular survival. Following internalization into phagosomes, intracellular bacteria interfere with phagosome maturation and inhibit phagosome–lysosome fusion, thereby preventing exposure to the degradative lysosomal environment. They also suppress the microbicidal mechanisms of macrophages, including the generation of reactive oxygen species (ROS), nitric oxide (NO), hydrolytic enzymes, and antimicrobial peptides. In addition, intracellular bacteria inhibit autophagic clearance, further enhancing their intracellular persistence. Many pathogens also promote macrophage polarization toward the anti-inflammatory M2 phenotype, which favors bacterial survival and persistence. Furthermore, intracellular bacteria reprogram host cellular metabolism and exploit host-derived metabolites to support their growth and replication.

**Figure 3:**
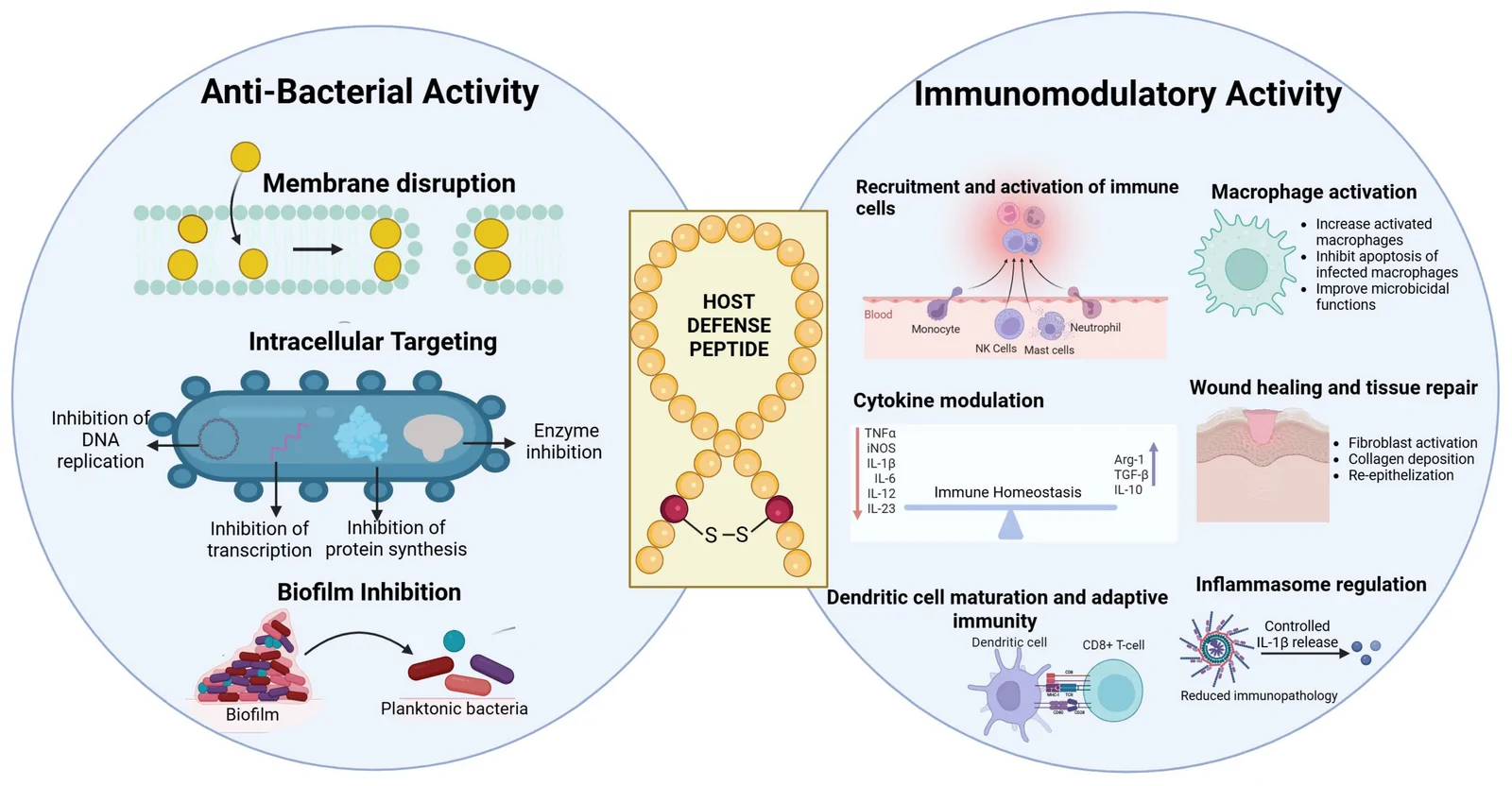
**Overview of the mechanisms of host defense peptides against intracellular bacterial infections**: Host defense peptides (HDPs) eliminate intracellular bacteria through a combination of direct antimicrobial activity and modulation of the host immune response. Their cationic, amphipathic structures enable HDPs to interact with bacterial membranes, leading to membrane disruption and bacterial death. In addition to membrane-targeting activity, HDPs interfere with essential intracellular bacterial processes, including DNA replication, transcription, protein synthesis, and enzymatic functions. Several HDPs also inhibit biofilm formation, thereby limiting bacterial persistence and antimicrobial tolerance. Beyond their direct antimicrobial effects, HDPs enhance host immune surveillance by promoting the recruitment of immune cells and augmenting the microbicidal functions of macrophages. HDPs further regulate cytokine production to control excessive inflammation while preserving effective antimicrobial immunity, thereby maintaining immune homeostasis. In addition, HDPs facilitate wound healing and tissue repair, contributing to the restoration of tissue integrity following infection.

**Figure 4:**
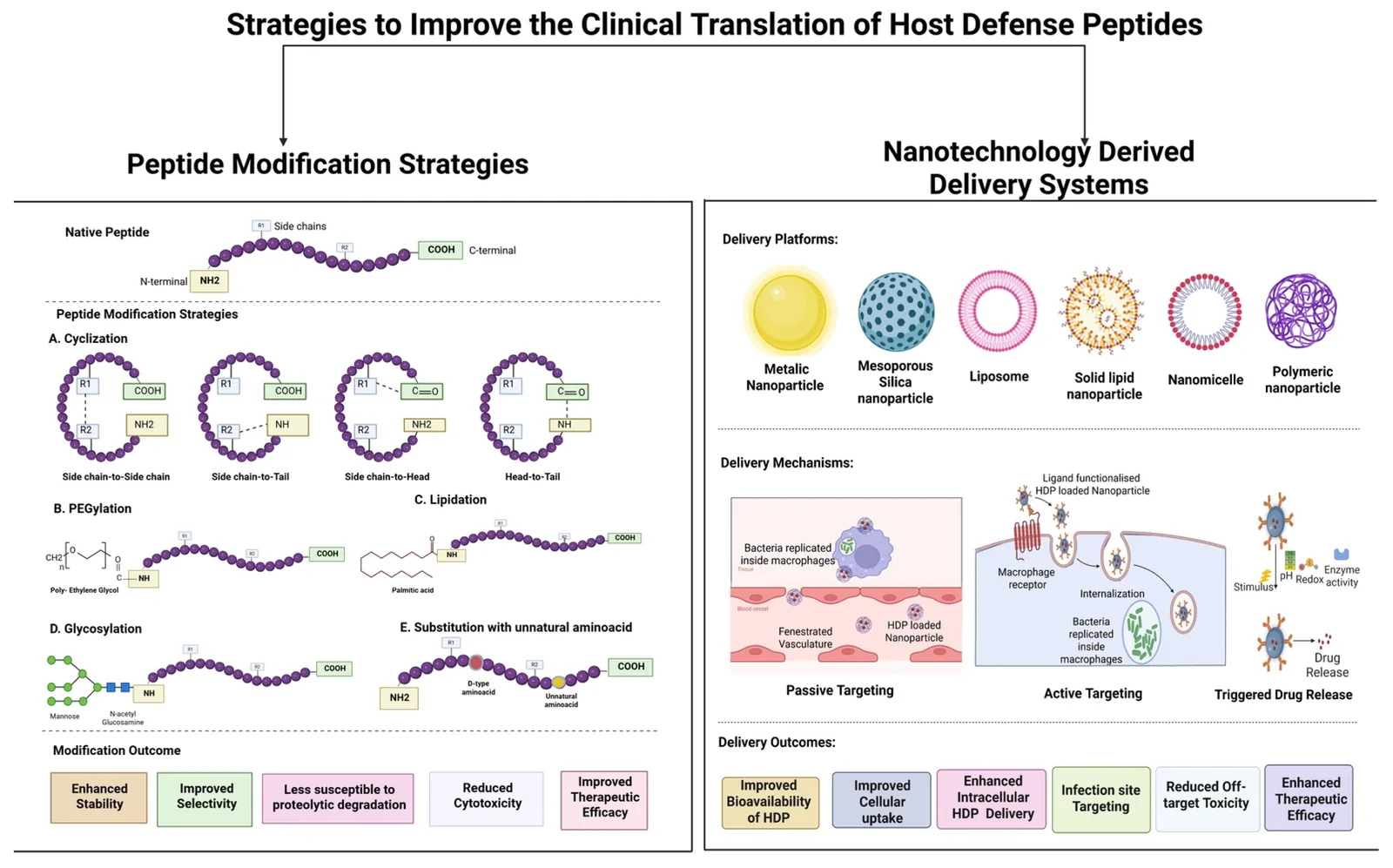
**Schematic overview of peptide modification and nanocarrier-based delivery strategies for enhancing the therapeutic potential of host defense peptides:** The left panel illustrates chemical modification strategies, including cyclization, PEGylation, lipidation, glycosylation, and incorporation of non-canonical amino acids, which enhance peptide stability and pharmacokinetic properties while reducing toxicity and improving therapeutic efficacy. The right panel depicts nanocarrier-based delivery systems, including nanoparticles, liposomes, and nano micelles, which facilitate efficient intracellular delivery and targeted accumulation of HDPs at infection sites, thereby improving peptide exposure, therapeutic efficacy, and safety.

**Table 1:** Clinically relevant intracellular bacteria, associated illness, host cell where it persists and conventional antibiotic therapy.

Intracellular Bacteria	Disease	Host Cell Localization	Conventional Therapy
**Obligate Intracellular Bacteria**			
*Chlamydia trachomatis* [21]	Genital and ocular infections	Epithelial cells in the genital, respiratory and ocular tracts	Doxycycline or Azithromycin
*Chlamydia pneumoniae* [22]	Respiratory tract infections	Respiratory epithelial cells, macrophages	Doxycycline or Azithromycin
*Rickettsia rickettsii* [23]	Rocky Mountain spotted fever	Monocytes/Macrophages	Doxycycline
*Coxiella burnetii* [24]	Q fever	Monocytes/Macrophages	Doxycycline
*Anaplasma phagocytophilum* [25]	Human granulocytic anaplasmosis	Neutrophils	Doxycycline
**Facultative intracellular Bacteria**			
*Mycobacterium tuberculosis* [26]	Tuberculosis	Macrophages, alveolar macrophages	Rifampicin + Isoniazid + Pyrazinamide + Ethambutol
*Mycobacterium leprae* [27]	Leprosy	Macrophages, Schwann cells	Dapsone + Rifampicin + Clofazimine
*Salmonella enterica* serovar Typhi [28]	Typhoid fever	Macrophages, monocytes	Ceftriaxone, Azithromycin, Fluoroquinolones
*Listeria monocytogenes* [29]	Listeriosis	Macrophages, epithelial cells, hepatocytes	Ampicillin/Gentamicin
*Brucella* spp. [30]	Brucellosis	Macrophages, dendritic cells	Doxycycline + Rifampicin
*Legionella pneumophila* [31]	Legionnaires’ disease	Alveolar macrophages	Azithromycin or Levofloxacin
*Yersinia pestis* [32]	Plague	Macrophages (early stage)	Streptomycin, Gentamicin
*Shigella flexneri* [33]	Bacillary dysentery	Macrophages, intestinal epithelial cells	Ciprofloxacin, Azithromycin
*Klebsiella pneumoniae* [34]	Pneumonia, sepsis, liver abscess	Macrophages, epithelial cells	Carbapenems, Cephalosporins, Aminoglycosides, depends on antibiotic susceptibility
*Staphylococcus aureus* [35]	Dermal infection, osteomyelitis, mastitis	Macrophages, Epithelial cells, Osteoblast, Endosome, Cytosol	Vancomycin, Linezolid, Daptomycin

**Table 4:** Overview of peptide engineering strategies to improve therapeutic value of HDPs.

Strategy	Design Modification	Major Outcome	Representative Examples
Rational peptide design and optimization	• Optimization of peptide sequence • Synthetic analogues	• Improved structural stability and antibacterial efficacy • Enhanced resistance to proteolytic degradation • Improved pharmacokinetic properties • Enhanced selectivity with reduced toxicity	• Mastoparan: Incorporation of the pentapeptide motif (FLPII) motif into the N-terminus of mastoparan enhanced host-defense and direct antibacterial activities while improving the safety profile [204] • RR4: Rational sequence optimization (maintenance of the α-helical conformation, incorporation of lysine and arginine residues) improved antibacterial, immunomodulatory, and antibiofilm activities, while reducing toxicity and enhancing proteolytic stability [207,208]
Backbone Cyclization	• Cyclization occurs through bonds involving the peptide backbone (Covalent N-to-C terminal linkage)	• Increases resistance to proteolytic degradation • Restrict conformational flexibility, helping the peptide maintain a biologically active conformation • Improve stability in biological fluids	• KR-12: Backbone cyclization improved the biological stability and antimicrobial activity of the peptide [210]
PEGylation	• Peptides are covalently conjugated with polyethylene glycol (PEG)	• PEGylation increases the molecular size of peptides, thereby reducing proteolytic degradation through steric hindrance and prolonging systemic circulation by delaying renal clearance	• Aurelin: PEGylated derivatives exhibited improved safety and antibacterial activity [211] • IDR-1018: N-terminal PEG6 conjugation enhanced solubility, reduced peptide aggregation, and improved safety while retaining immunomodulatory activity [212]
Lipidation	• Conjugation of fatty acids to peptides	• Lipidation promotes reversible binding to serum albumin • Reduces enzymatic degradation and delays renal clearance	• HDP B1: N-terminal conjugation with C6 and C8 fatty acids enhanced microbicidal activity and improved biocompatibility and stability compared with the native peptide biocompatibility and stability than the native peptide [213]
Substitution of D-amino acids/other non-canonical amino acids	• Replacement of natural L-amino acids with D-amino acids or non-canonical amino acids	• Alters both the peptide backbone and side-chain architecture • Reducing susceptibility to enzymatic degradation and enhancing resistance to proteases	• UP09: Substitution of selected L-Lys residues with homoarginine or α-aminoisobutyric acid improved proteolytic stability while maintaining biological activity and an acceptable safety profile *in vitro* and *in vivo* [215]

**Table 5:** Representative examples of drug delivery systems for host defense peptides.

Delivery System	Model Peptide	Features	Outcome
**Nanoparticle** **-based delivery systems**			
Gold nanoparticles	VG16KRKP	VG16KRKP peptide is tagged on gold nanoparticle (Au-VG16KRKP) against Salmonella infection	• Effective Intracellular penetration into macrophages and epithelial cells • Potent bacteriolytic activity against intracellular *Salmonella Typhimurium* *in vitro* and *in vivo* • Pharmacologically safe *in vivo* [217]
Silver nanoparticles	H2N-IFH-COOH (IFH) composed of three amino acids: isoleucine, phenylalanine, and histidine	Silver-peptide colloidal nanohybrids prepared using amphiphilic tripeptide building block (IFH) and silver nitrate (AgNO_3_)	• Antibacterial activity against Gram-negative and Gram-positive bacterial strains • Enhanced biocompatibility [220]
Mesoporous silica nanoparticles	G818	G818 along with 2-(1,8-naphthyridin-2-yl) phenol (2-NP) (macrophage polarization modulator) loaded on dendritic mesoporous silica nanoparticle system)	• Dendritic mesoporous silica nanoparticle system functions as a molecular sprayer that releases the drug to the acidic microenvironment of infection sites • Sustained antibacterial activity with macrophage regulation and bone formation efficacy in the treatment model of bone infection [222]
**Liposomes and solid lipid nanoparticles**			
Liposome	FWR33	Synthetic peptide FWR33 is coupled with an amphipathic α-helical fusion peptide and surface-conjugated to liposomes to form functional antimicrobial liposomes	• Improve antibacterial performance against *Escherichia coli* and *Staphylococcus aureus* • Non-toxic to mammalian cells • Prevented enzymatic hydrolysis of the peptide to enhance its stability [223]
Nano micelles	DP7	DP7 is conjugated with cholesterol to form an amphiphilic conjugate (DP7-C), which self-assembles into stable nanomicelles in aqueous solution	• Less hemolytic activity than parent peptide upon IV administration • Enhanced the antibacterial and immunomodulatory activity in multiple *in vivo* models of systemic infectious disease [225]
lipid nanoparticles	TAT peptide	• TAT peptide is conjugated with fatty acid chains of varying lengths to form lipidated analogs (TAT-lipids). • Optimal TAT-lipid, C16-TAT, with better membrane-disruptive and cell-penetrating capacities was selected and incorporated into MC3 Lipid Nano Particles to generate AMP-derived LNPs (TAT-MC3 LNPs)	• Rapid uptake and efficient delivery of peptide to pulmonary cells • Better clearance of intracellular bacteria, *Staphylococcus aureus* and *Pseudomonas aeruginosa* enhanced its hemocompatibility and cytocompatibility • No detectable hepatic or pulmonary toxicity was observed following intravenous administration [227]
**Polymeric nanoparticles**			
Poly (lactide-co-glycolide) (PLGA nanoparticles	Esculentin (1-21) or Esculentin (1-21)-1c	PLGA nanoparticles for lung delivery of Esculentin peptides	• Improved efficacy in inhibiting *Pseudomonas aeruginosa* growth *in vitro* and *in vivo* • Single intratracheal administration of formulation resulted in a 3-log reduction in pulmonary bacterial burden up to 36 h in a mouse model of *Pseudomonas aeruginosa* lung infection [229]
Surface functionalization of PLGA nanoparticles	MSI-78	Conjugation of MSI-78 onto the surface of PLGA nanoparticles	• Peptide exposure on the nanoparticle surface • Direct interaction with bacteria improved infection-site targeting • Rapid onset of antibacterial activity against *Pseudomonas aeruginosa* and *Staphylococcus aureus* [230]
Chitosan nanoparticles	LL-37, microcin J25,3W2	Encapsulation of peptides in Chitosan nanoparticles	• Enhanced peptide stability • Sustained drug release • Improved cellular compatibility • Enhanced antibacterial efficacy [232,233,234]
**Macrophage-targeted delivery strategies**	Tuftsin, TAT	The macrophage-targeting peptide tuftsin (TKPR) and the cell-penetrating peptide TAT are linked by a cathepsin-cleavable Val-Cit linker. Self-assembly of the peptide into nanomicelles Rifampicin was subsequently encapsulated within the hydrophobic core of the nanomicelles	• Tuftsin moiety binds to the neuropilin-1 (NP-1) receptor on macrophages; the nanomicelles were internalized through receptor-mediated endocytosis • Val-Cit linker was selectively cleaved by lysosomal cathepsins, exposing the TAT–6-valine fragment and facilitating intracellular release of rifampicin • The released peptide fragment and rifampicin acted synergistically to eliminate intracellular *Staphylococcus aureus* [236]

**Table 6:** Cell-penetrating peptides with anti-bacterial activity.

Cell-Penetrating Peptide	APD ID	Source	Sequence	Target Organism
Crotamine [244,245]	AP01650	Natural, venom, *Crotalus durissus terrificus*	YKQCHKKGGHCFPKEKICLPPSSDFGKMDCRWRWKCCKKGSG	*Escherichia coli*
Engraulisin [246]	AP03829	Synthetic	RLFRHRFKRVLRL	*Staphylococcus aureus*, MRSA
LDP-NLS [247]	AP03813 (for LDP)	Synthetic- Prepared by fusing LDP derived from Latarcin 1 toxin with nuclear localization sequence (NLS)	KWRRKLKKLRPKKKRKV	Methicillin-resistant *Staphylococcus aureus* (MRSA)
PenShuf [248]	AP03810	Synthetic	RWFKIQMQIRRWKNKK	*Staphylococcus aureus* and *Escherichia coli*
Pep-1 [249]	AP03815	Synthetic	*N*-acetyl-KETWWETWWTEWSQPKKKRKV-OH	*Chlamydia trachomatis*
[I11S] Tachyplesin I [250]	AP03817	Synthetic	KWCFRVCYRGSCYRRCR	*Escherichia coli*
TAT (47–58) [251]	AP03814	Derived from the HIV-1 regulatory protein Tat	YGRKKRRQRRRD	Methicillin-resistant *Staphylococcus aureus*
Tpk [252]	AP03809	Synthetic	VRRFkWWWkFLRR k refers to NLys[H2N-(CH2)4-NH-CH2-COOH]	*Staphylococcus aureus*

Details of the CPPs were also retrieved from the Antimicrobial Peptide Database (APD6), with the corresponding APD accession numbers provided for each peptide [146].

## Data Availability

No new data were created or analyzed in this study. Data sharing is not applicable to this article.
